# Disease Aggravation With Age in an Experimental Model of Multiple Sclerosis: Role of Immunosenescence

**DOI:** 10.1111/acel.14491

**Published:** 2025-02-02

**Authors:** María Dema, Herena Eixarch, Arnau Hervera, Mireia Castillo, Luisa M. Villar, Xavier Montalban, Carmen Espejo

**Affiliations:** ^1^ Servei de Neurologia, Centre d'Esclerosi Múltiple de Catalunya (Cemcat), Vall d'Hebron Institut de Recerca (VHIR) Hospital Universitari Vall d'Hebron Barcelona Spain; ^2^ Universitat Autònoma de Barcelona Bellaterra Spain; ^3^ Departmento de Inmunología Hospital Universitario Ramón y Cajal, Instituto Ramón y Cajal de Investigación Sanitaria (IRYCIS) Madrid Spain

**Keywords:** adaptive immunity, aging, experimental autoimmune encephalomyelitis, immunosenescence, innate immunity, multiple sclerosis, neurodegeneration, neuroinflammation

## Abstract

The onset of multiple sclerosis (MS) in older individuals correlates with a higher risk of developing primary progressive MS, faster progression to secondary progressive MS, and increased disability accumulation. This phenomenon can be related to age‐related changes in the immune system: with age, the immune system undergoes a process called immunosenescence, characterized by a decline in the function of both the innate and adaptive immune responses. This decline can lead to a decreased ability to control inflammation and repair damaged tissue. Additionally, older individuals often experience a shift toward a more pro‐inflammatory state, known as inflammaging, which can exacerbate the progression of neurodegenerative diseases like MS. Therefore, age‐related alterations in the immune system could be responsible for the difference in the phenotype of MS observed in older and younger patients. In this study, we investigated the effects of age on the immunopathogenesis of experimental autoimmune encephalomyelitis (EAE). Our findings indicate that EAE is more severe in aged mice due to a more inflammatory and neurodegenerative environment in the central nervous system. Age‐related changes predominantly affect adaptive immunity, characterized by altered T cell ratios, a pro‐inflammatory Th1 response, increased regulatory T cells, exhaustion of T cells, altered B cell antigen presentation, and reduced NK cell maturation and cytotoxicity. Transcriptomic analysis reveals that fewer pathways and transcription factors are activated with age in EAE. These findings allow us to identify potential therapeutic targets specific to elderly MS patients and work on their development in the future.

## Graphical abstract

Age‐related differences in EAE outcomes stem from combined peripheral and CNS factors. Key peripheral adaptive immunity changes include altered naïve/memory T cell ratios, Th1 skewing, increased suppressive Tregs, CD4+ T cell exhaustion, enhanced B cell antigen presentation, and reduced NK cell cytotoxicity. Aged EAE mice also exhibit heightened CNS inflammation and neurodegeneration, worsening disease severity. Peripheral transcriptomic profiling highlights potential therapeutic targets for MS patients over 50, addressing age‐associated immune and neurodegenerative contributions to increased disability.

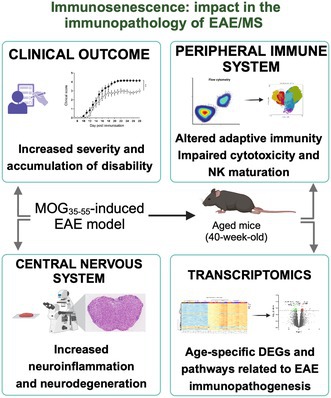

## 1 Introduction

Multiple sclerosis (MS) is a chronic inflammatory, demyelinating, and neurodegenerative disease of the central nervous system (CNS). MS affects approximately 2.9 million people worldwide with a prevalence of 1 in every 300–3000 people and it is the primary cause of non‐traumatic disability in young adults. MS typically initiates in young adults (from 20 to 40 years of age), however, MS can also initiate in younger (under 18) and older (over 50) people. It is more prevalent in women than men with an incidence ratio of 1.9–3.5:1, depending on the geographical region (Federation 2020). Most patients initiate with a relapsing–remitting MS (RRMS) (85%), the majority of which accumulate disability progressively with time leading to the development of secondary progressive MS (SPMS). A minority of patients present a progressive disease course from the onset, known as primary progressive MS (PPMS) (15%). There are growing evidence that point to aging as a significant factor influencing the course of MS: RRMS patients over 50 years old tend to develop SPMS despite the time of evolution of the disease, in addition, the debut at an older age associates with increased risk of presenting PPMS, earlier conversion to SPMS and greater disability accumulation (Scalfari et al. 2011). This is of special relevance taking into account that the number of elderly MS patients is growing mainly due to the increase in life expectancy of the general population (WHO 2022) and the availability of highly effective disease‐modifying treatments (DMTs). The fact that MS features differ in patients over 50 compared to younger patients reflects different underlying immune mechanisms in both populations and that changes in the immune system related to aging may have a relevant role in disease progression.

Immunosenescence is defined as a progressive and gradual age‐related decline of the immune function, resulting in higher susceptibility to infections, reduced response to vaccines (Pera et al. 2015), and higher prevalence of autoimmunity (Goronzy and Weyand 2012) and neurodegenerative disorders (Costantini, D'Angelo, and Reale 2018). Age‐related changes in the adaptive immune system are well established, while those happening in the innate immunity are not well understood yet. These events that happen in the aged immune system affect not only the functionality but also the number and frequency of immune cells, which can result in the accumulation of unusual immune cell populations (Dema et al. 2021). Moreover, immunosenescence features have been described to appear earlier in patients diagnosed with autoimmune diseases like systemic lupus erythematosus, rheumatoid arthritis (RA), autoimmune thyroid disease (AITD), and Sjögren syndrome (SjS) (Thewissen et al. 2007). Although there are some studies that address the impact of immunosenescence on MS patients and on experimental autoimmune encephalomyelitis (EAE) (Bolton and Smith 2018), there is still a long way to explore.

In the present study, we aimed to comprehensively unveil the changes that age induces in the immunopathogenesis of MS through the study of a well‐established experimental model of the disease, myelin oligodendrocyte glycoprotein (MOG)‐induced EAE.

## 2 Results

### 2.1 EAE Clinical Outcome is More Severe With Age

**FIGURE 1:**
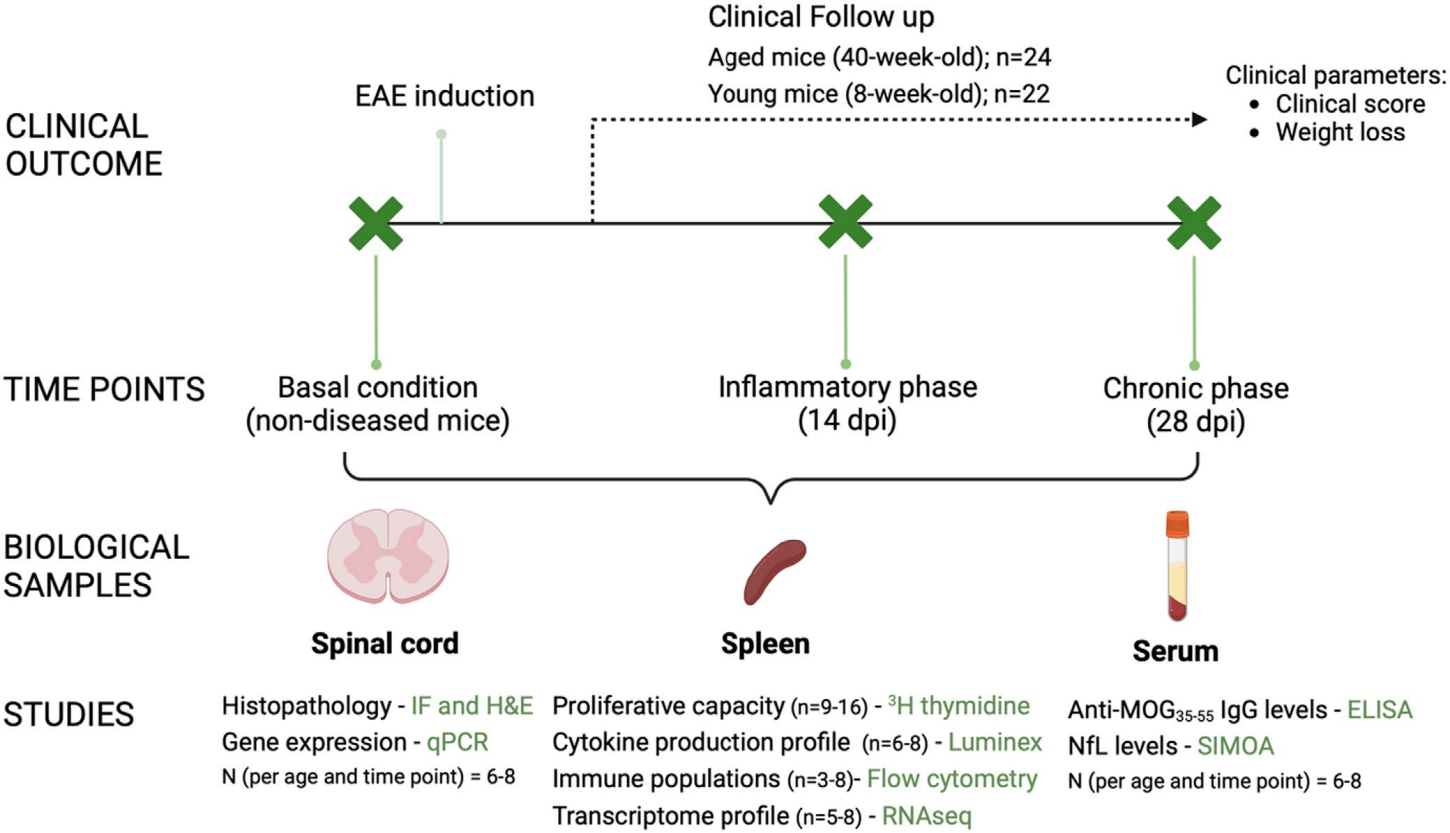
Overview of the study design and sampling strategy. The study design evaluated clinical outcomes, gene expression, and immunological differences at three key time points: basal (pre‐EAE induction), inflammatory phase (14 dpi), and chronic phase (28 dpi). Young (8‐week‐old) and aged (40‐week‐old) mice were included and were followed up for 28 days. Clinical parameters were studied on 24 aged mice and 22 young mice. Numbers of mice analyzed per technique and sample type are indicated. All clinical, immunologic, and transcriptomic analyses were performed on incident mice that reached the clinical endpoint. Biological samples were collected, including the spinal cord, spleen, and serum at each time point. Key analyses included histopathology (immunofluorescence and hematoxylin and eosin staining) and qPCR for spinal cord; immune profiling, cytokine production (Luminex), and transcriptomic profiling (RNAseq) for spleen; and biomarker quantification (anti‐MOG_35–55_ IgG levels by ELISA and neurofilament light chain (NfL) levels by SIMOA) for serum. For the analysis of gene expression and immunological variability, we employed linear mixed models to evaluate cytokine production and immune cell populations. For transcriptomic analysis (RNAseq), principal component analysis (PCA) was performed to evaluate the clustering of samples within age groups. DPI, days post‐induction; EAE, experimental autoimmune encephalomyelitis; H&E, hematoxylin and eosin; IF, immunofluorescence.

For this study, we used 8‐week‐old (young mice; equivalent to 20 years old in humans) and 40‐week‐old (aged mice; equivalent to 50 years old in humans) females. The study design is detailed in Figure 1.

**FIGURE 2:**
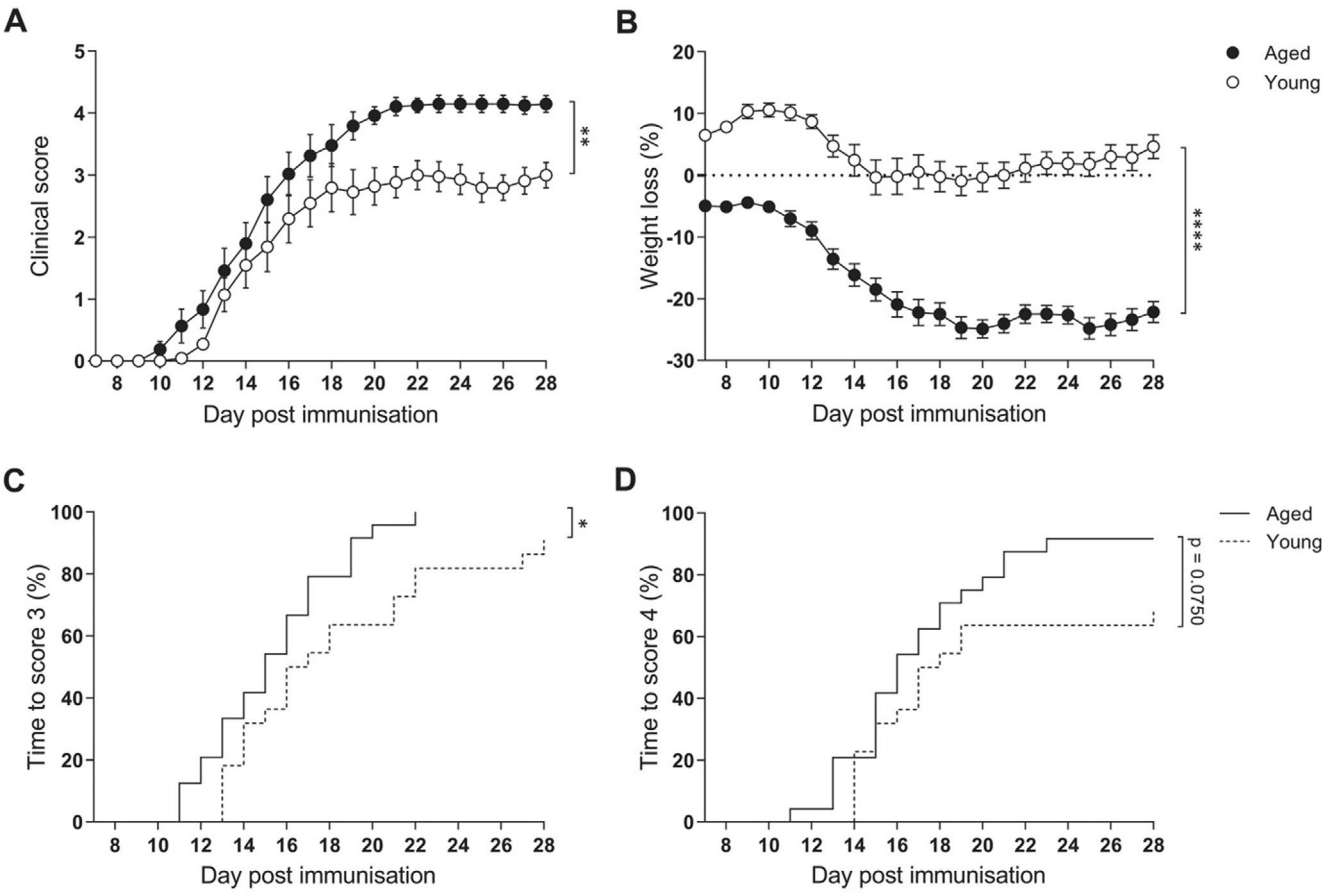
EAE clinical outcomes become more severe with age. (A) Clinical course. (B) Weight loss. (C) Time to reach a score of 3 (mild tetraparesis). (D) Time to reach a score of 4 (tetraparesis) in young and aged mice. Data represent three independent experiments with *n* = 22 for young mice and *n* = 24 for aged mice at 28 dpi. Only incident mice were included in the analysis. Variables were analyzed using the MIXED model and statistical significance correction for multiple comparisons was performed with Bonferroni in A‐B and Log‐rank test in C, D. Data are expressed as the mean ± SEM in A, B and as survival curves in C, D. *p < 0.05; **p < 0.01; ****p < 0.0001.

We first aimed to elucidate whether age influences the clinical outcome of EAE, for that the disease was induced in young and aged mice. EAE was more severe in aged mice (Movie S1) compared to young mice (Movie S2), indeed, aged mice reached a higher maximum clinical score (Table S1), accumulated more disability (Figure 2A), and lost more weight during disease progression (Figure 2B) than young counterparts. Moreover, aged mice reached mild tetraparesis earlier than young mice (Figure 2C) and also tended to reach tetraparesis earlier (Figure 2D), indicating that age influences the clinical outcome of the disease.

### 2.2 Local Inflammation and Neurodegeneration are Exacerbated With Age

**FIGURE 3:**
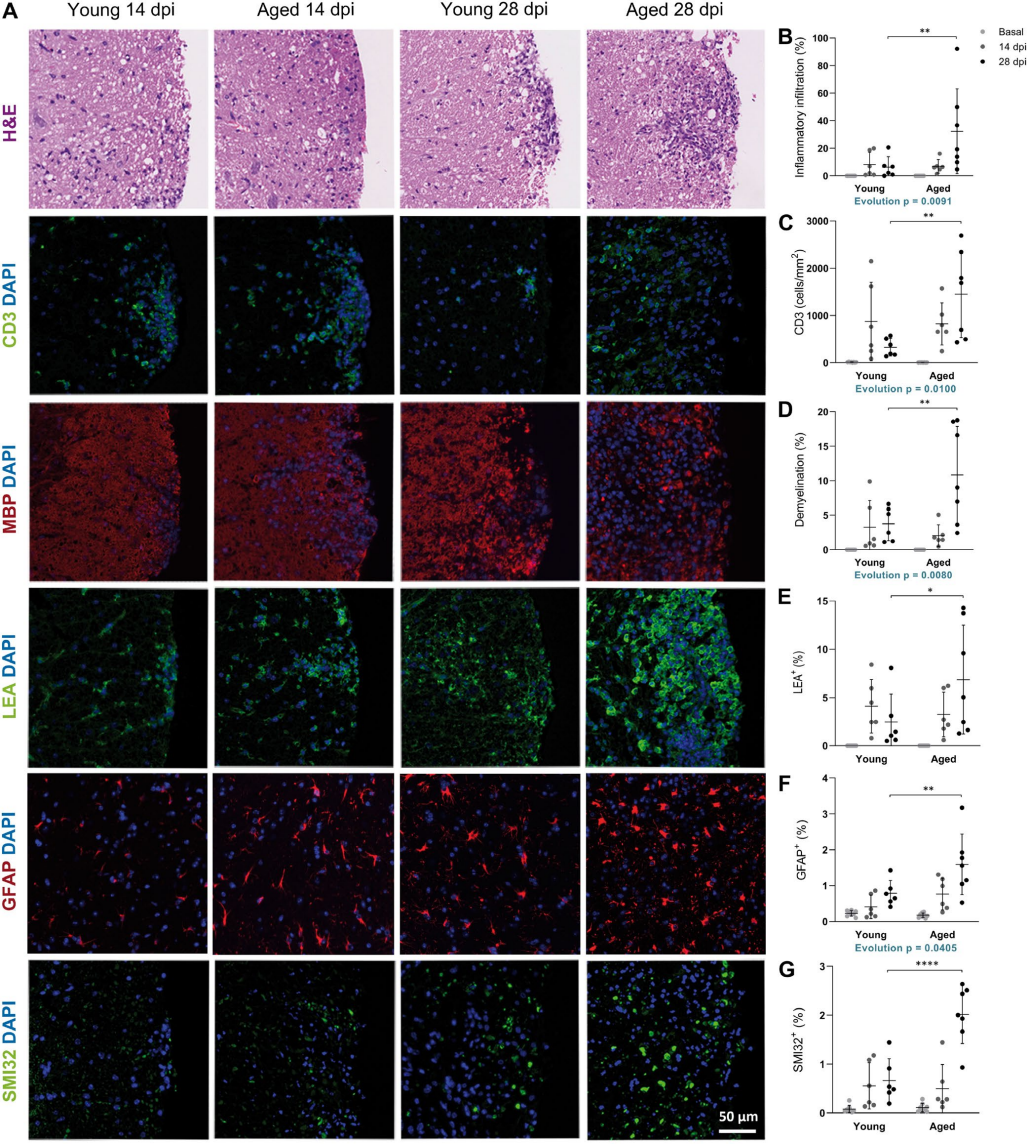
CNS histopathological analysis reflects aged‐related inflammation and neurodegeneration in EAE. (A) Representative images of histopathological analysis in the white matter of the spinal cord at 14 and 28 dpi in young and aged mice. (B–G) Quantification of inflammatory infiltration (B), infiltrating T lymphocytes (C), demyelination (D), reactive microglia/infiltrating macrophages (E), reactive astroglia (F), and axonal damage (G) in the white matter of the spinal cord at basal (non‐immunized mice), 14 and 28 dpi in young and aged mice. Data represent an individual experiment with *n* = 8 (basal), *n* = 6 (14 dpi), and *n* = 6 (28 dpi) for young mice and *n* = 8 (basal), *n* = 6 (14 dpi), and *n* = 7 (28 dpi) for aged mice. Only incident mice that reached the endpoint were included in the analysis. Variables were analyzed using the MIXED model and statistical significance correction for multiple comparisons was performed with Bonferroni adjustment. Data are expressed as the mean ± SD. *p < 0.05; **p < 0.01; ****p < 0.0001. GFAP, glial fibrillary acidic protein; H&E, hematoxylin and eosin; LEA, *Lycopersicon esculetum*; MBP, myelin basic protein; SMI32, neurofilament H non‐phosphorylated.

We then evaluated the effect of age on inflammation and neurodegeneration in the spinal cord of aged and young EAE mice, in both the inflammatory and chronic phases of the disease. According to the age‐related increase in the severity of the clinical outcome, histopathological analysis revealed that, in the chronic phase of EAE, aged mice presented an increase in inflammatory infiltration (Figure 3A,B), infiltrating T lymphocytes (Figure 3A,C), demyelination (Figure 3A,D), reactive microglia/infiltrating macrophages (Figure 3A,E), and reactive astroglia (Figure 3A,F). In addition, aged mice also presented more axonal damage during the chronic phase of EAE (Figure 3A,G) that correlated with higher neurofilament light chain (NfL) levels in serum (Figure S1), confirming higher neurodegeneration in EAE with age. Although no differences were observed at the inflammatory phase of EAE, evolution patterns along the disease course were also different for inflammatory infiltration, infiltrating T lymphocytes, demyelination, and reactive astroglia, indicating that the local inflammatory and neurodegenerative events during EAE progress differently with age. Moreover, no differences were detected in the histopathological analyses comparing non‐immunized young and aged mice (basal condition, Figure 3B–G), suggesting that non‐immunized aged mice did not display an enhanced proinflammatory or neurodegenerative state. This indicates that the accumulation of infiltration and CNS damage observed during the chronic phase of the disease in aged mice results from distinct or intensified immunopathogenic processes.

**FIGURE 4:**
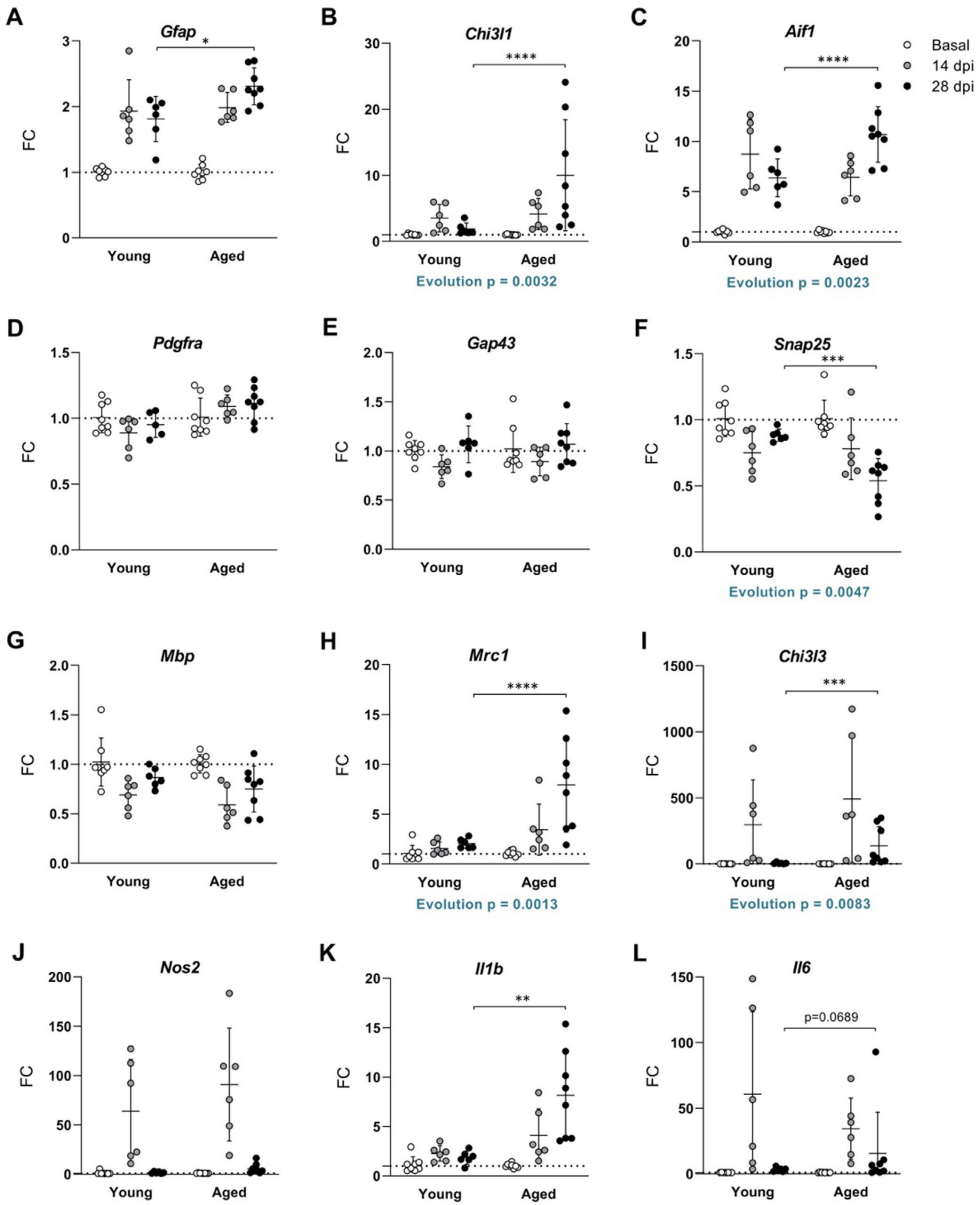
CNS gene expression analysis confirms aged‐related inflammation and neurodegeneration in EAE. (A–L) Gene expression of *Gfap* (A), *Chi3l1* (B), *Aif1* (C), *Pdgfra* (D), *Gap43* (E), *Snap25* (F), *Mbp* (G), *Mrc1* (H), *Chi3l3* (I), *Nos2* (J), *Il1b* (K), and *Il6* (L) in the spinal cord at basal (non‐immunized mice), 14 and 28 dpi in young and aged mice. Values were normalized against *Gapdh*. The dotted line indicates an FC value of 1. Data represent an individual experiment with *n* = 8 (basal), *n* = 6 (14 dpi), and *n* = 6 (28 dpi) for young mice and *n* = 8 (basal), *n* = 6 (14 dpi), and *n* = 8 (28 dpi) for aged mice. Only incident mice that reached the endpoint were included in the analysis. Variables were analyzed using the GLIMMIX model and statistical significance correction for multiple comparisons was performed with Bonferroni adjustment. Data are expressed as the mean ± SD. *p < 0.05; **p < 0.01; ***p < 0.001; ****p < 0.0001. FC, fold change.

We further confirmed the most extensive inflammatory and neurodegenerative environment in aged mice by gene expression analysis of the spinal cord. Inflammation and neurodegeneration were more pronounced in aged than in young mice during the chronic phase of EAE. For instance, the expression of *Gfap* (Figure 4A) and *Chi3l1* (Figure 4B), as well as *Aif1* (Figure 4C) were found to increase in aged mice, indicating a higher reactivity of astrocytes. Age also affected the capacity of neural regeneration, since the expression of the gene *Snap25* was decreased in EAE aged mice (Figure 4F). The expression of the mannose receptor *Mrc1* (Figure 4H) and *Chi3l3* (Figure 4I) were also increased in aged mice during the chronic phase of EAE, indicating an attempt to resolve inflammation and to regenerate the damaged tissue, probably as a consequence of the pro‐inflammatory and neurodegenerative environment that persists in the CNS of aged mice. However, no age‐related differences were observed in the expression of the *Pdgfra* oligodendrogenesis marker (Figure 4D), *Gap43* neuroregeneration marker (Figure 4E), and *Mbp* myelination marker (Figure 4G). It should be highlighted that the expression of the innate immunity cytokine *Il1b* significantly increased in aged mice (Figure 4K) and *Il6* was also elevated, although differences did not reach statistical significance (Figure 4L), but *Nos2* oxidative stress marker (Figure 4J) expression did not change. Altogether indicates that the severity of the disease in aged mice is more likely due to inflammatory events than to oxidative damage or a defect in the myelinating capacity of oligodendrocytes. Additionally, the evolution pattern along the disease was also different between ages for the expression of *Chi3l1*, *Aif1*, *Snap25*, *Mrc1*, and *Chi3l3* genes, confirming that the molecular signature in EAE progression is different with age.

### 2.3 Peripheral Immune Response Reveals Age‐Related Changes in EAE

**FIGURE 5:**
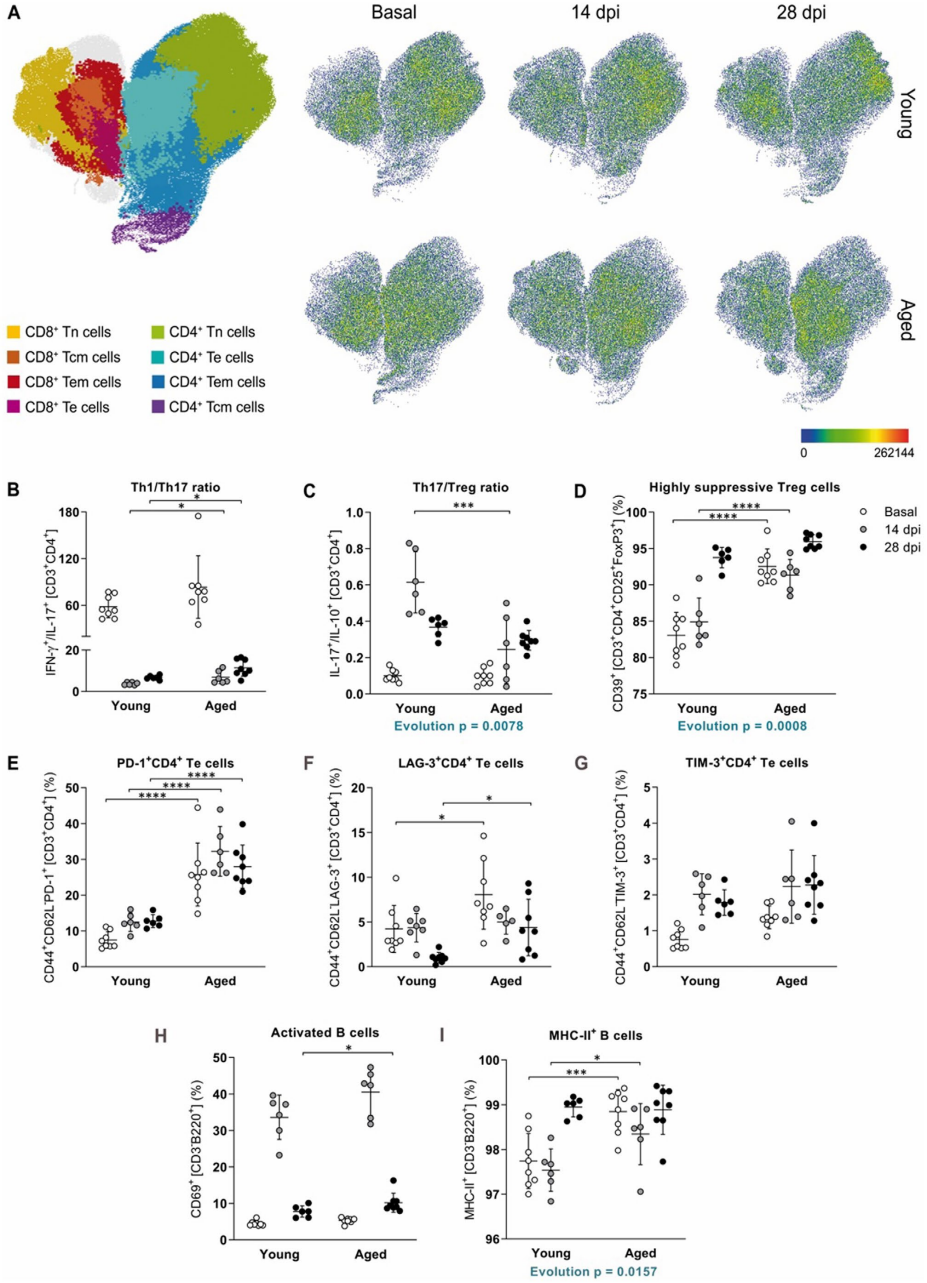
Peripheral adaptive immune cell populations reveal age‐related changes in EAE. (A–I) T cell differentiation (A), Th1/Th17 ratio (B), Th17/Treg ratio (C), highly suppressive Treg cells (D), PD‐1^+^ (E), LAG‐3^+^ (F), TIM‐3^+^CD4^+^ Te cells (G), activated B cells (H), and MHC‐II^+^ B cells (I) at basal (non‐immunized mice), 14 and 28 dpi in young and aged mice. Data represent an individual experiment with *n* = 8 (basal), *n* = 6–7 (14 dpi), and *n* = 6–8 (28 dpi) for young mice and *n* = 8 (basal), *n* = 5–6 (14 dpi), and *n* = 8 (28 dpi) for aged mice. Only incident mice that reached the endpoint were included in the analysis. Variables were analyzed using the MIXED model and statistical significance correction for multiple comparisons was performed with Bonferroni adjustment. Data are expressed as UMAP in A and the mean ± SD in B‐I. *p < 0.05; ***p < 0.001; ****p < 0.0001. LAG‐3, lymphocyte activating gene 3; MHC, major histocompatibility complex; PD‐1, programmed cell death protein 1; Tcm, central memory T; Te, effector T; Tem, effector memory T; Th, helper T; TIM‐3, T cell immunoglobulin and mucin domain‐containing protein‐3; Tn, naïve T; Treg, regulatory T.

We also studied the potential changes in the peripheral immune cell populations and response in aged EAE mice. The majority of age‐related changes occurred in cells of the adaptive immunity, more specifically in the T cell compartment. Our results showed an imbalance in naïve and memory T cells in both the CD4^+^ and CD8^+^ T cell compartments in EAE with age, since, as EAE progressed, the frequency of naïve T (Tn) cells was contracted and, at the same time, there was an accumulation of effector CD4^+^ T cell populations, specifically effector T (Te), effector memory T (Tem), and activated CD4^+^ T cells. In addition, CD8^+^ Te, central memory T (Tcm), and activated T cells accumulated in aged EAE mice in the chronic phase (Figure 5A). The evolution pattern along EAE, this is how the frequency of a given population changed along EAE progression, were different between ages for CD4^+^ Tem and CD8^+^ Tn. In addition, no differences were observed in terminally differentiated effector T (Ttde) cells (Table S2).

Focusing on CD4^+^ Te cells, these cells play pivotal roles in orchestrating immune responses and not only are they important in protective immunity, but also have a key role in the development of autoimmunity. Because we found that major changes related to age occurred in the CD4^+^ T cell compartment and more specifically in CD4^+^ Te cells, we next characterized helper T (Th) responses by their cytokine production: Th1 (IFN‐γ^+^), Th2 (IL‐4^+^), Th17 (IL‐17^+^), and regulatory T (Treg) (IL‐10^+^) responses. Aged mice presented an enhanced production of both pro‐inflammatory and anti‐inflammatory cytokines. However, in aged mice, we observed an increase in the Th1/Th17 ratio (Figure 5B) and a decrease in the Th17/Treg ratio (Figure 5C) during the inflammatory phase of EAE. The increase in the Th1/Th17 ratio is maintained in the chronic phase of EAE, reflecting that disease worsening might be driven by the Th1 rather than the Th17 response. Although no differences were found in frequencies of FoxP3^+^ Treg cells, we analyzed the expression of the ectonucleotidase CD39, a functional marker of highly suppressive Treg cells. The frequency of CD39 expression on Treg cells was already elevated in non‐diseased aged mice (basal condition) and was maintained high compared to young mice in the inflammatory phase of EAE (Figure 5D). The evolution pattern of Th17/Treg ratio and highly suppressive Treg cells were different between ages along EAE (Table S3).

Together with Treg cells, there are other regulators of the immune response such as programmed cell death protein 1 (PD‐1), lymphocyte activating gene 3 (LAG‐3), and T cell immunoglobulin and mucin domain‐containing protein‐3 (TIM‐3) known as immune checkpoint inhibitors, whose function is to end an ongoing immune response; however, they are known to be accumulated with age, contributing to a malfunctioning of the senescent immune system, thus they are considered exhaustion markers in the context of aging. Our results showed again major age‐related exhaustion changes in the CD4^+^ T cell compartment. Taking into account the well‐described accumulation of T cells expressing immune checkpoint/exhaustion markers in the context of immunosenescence, it is not surprising that the percentages of PD‐1^+^ and Lag‐3^+^ CD4^+^ Te cells were higher in non‐diseased aged mice compared to their non‐diseased young counterparts. In addition, the higher frequency of PD‐1^+^ CD4^+^ Te cells was maintained elevated in aged EAE mice along the progression of the disease. On the other hand, there was an accumulation of these cells expressing LAG‐3 during the chronic phase of EAE in aged mice (Figure 5E–G; Table S4). Senescent cells are also characterized as lacking CD28 expression and accumulating natural killer (NK) receptors such as natural killer group 2D (NKG2D). Therefore, we also studied whether T cells acquired a senescent phenotype in EAE‐aged mice. Although we did not observe differences in the expression of CD28 and NKG2D in either CD4^+^ or CD8^+^ T cells, the frequency of the senescent populations presented a different evolution pattern along disease progression between ages (Table S5).

We detected the main differences in the activation status of B cells. Activated B cells were elevated in the chronic phase in aged mice (Figure 5H). Moreover, B cells from aged mice already presented increased expression of major histocompatibility complex (MHC)‐II in the basal condition (non‐immunized aged mice), and were found increased respect to young counterparts in the inflammatory phase of EAE (Figure 5I) and followed a different evolution pattern between ages (Table S6). Taking into account the observed increase in the activation status of B cells with age, we measured anti‐MOG_35‐55_ IgG autoantibody production in serum but no differences were found between ages in EAE (Figure S2). Taken together, these findings indicate that B cells from non‐diseased aged mice are more inclined towards antigen presentation, thereby potentially enhancing T cell activation in the context of a T cell‐mediated disease such as EAE.

**FIGURE 6:**
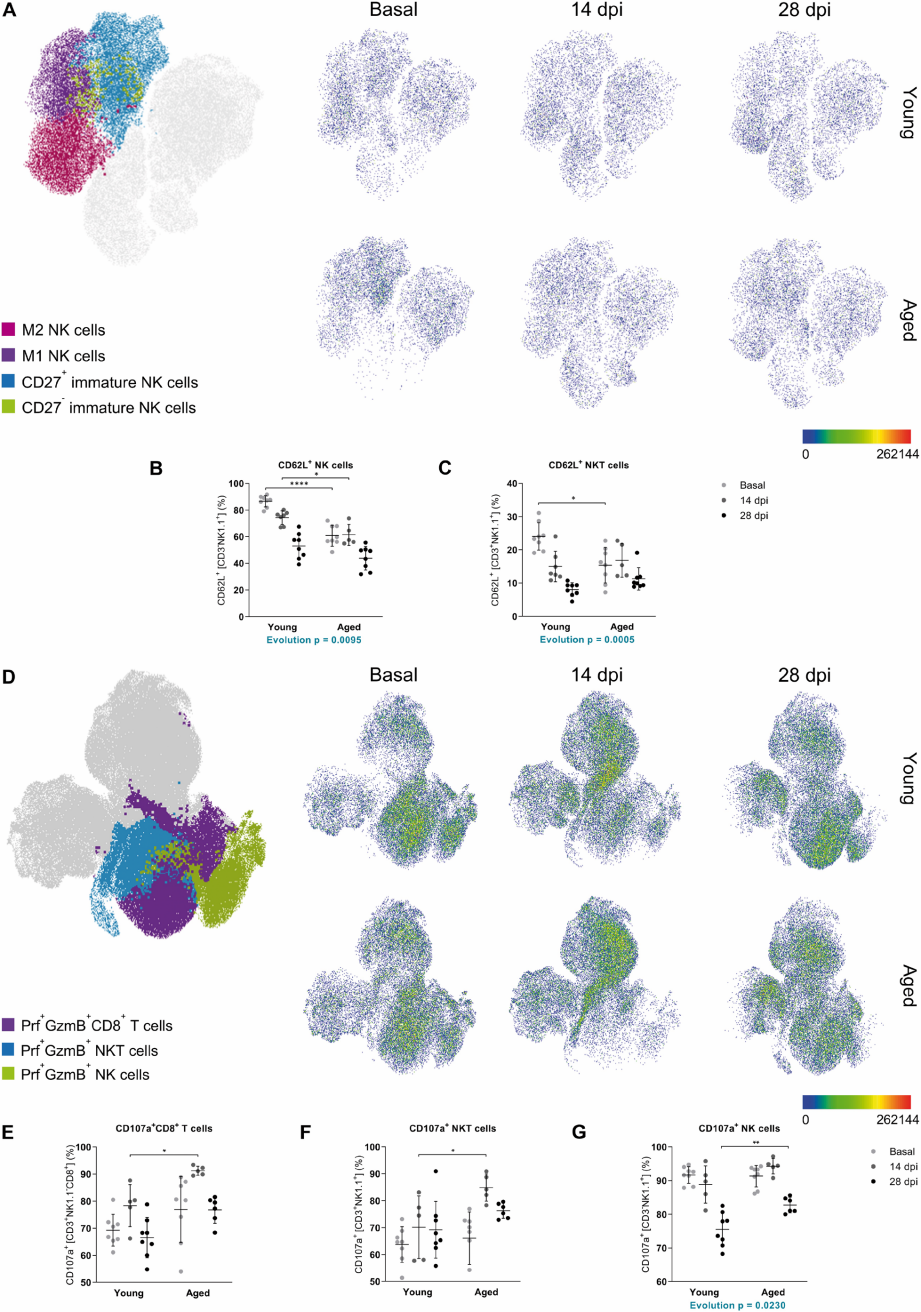
Peripheral innate immune cell populations reveal age‐related changes in EAE. (A–G) NK cell maturation (A), cytotoxic NK cells (B), cytotoxic NKT cells (C), CD8^+^ T, NKT and NK cell cytotoxicity (D), CD8^+^ T (E), NKT (F) and NK (G) cell degranulation at basal (non‐immunized mice), 14 and 28 dpi in young and aged mice. Data represent an individual experiment with *n* = 7–8 (basal), *n* = 3–7 (14 dpi), and *n* = 8 (28 dpi) for young mice and *n* = 8 (basal), *n* = 5 (14 dpi), and *n* = 6–8 (28 dpi) for aged mice. Only incident mice that reached the endpoint were included in the analysis. Variables were analyzed using the MIXED model and statistical significance correction for multiple comparisons was performed with Bonferroni adjustment. Data are expressed as UMAP in A and D and the mean ± SD in B‐C and E‐G. *p < 0.05; **p < 0.01; ****p < 0.0001. GzmB, granzyme B; NK, natural killer; NKT, natural killer T; Prf, perforin.

We found fewer age‐related changes in innate immunity. Although total numbers of the majority of innate immune cell populations like dendritic cells (DCs), neutrophils, and macrophages expressing Mrc1 did not change significantly (Table S7), we found that main age‐related differences were occurring in the NK and NKT cell compartments. We studied the percentage of total NK and NKT cells and their state of activation. Our results showed that the total population of NK cells was diminished and, contrarily, NKT cells were increased in non‐diseased aged mice and in the chronic phase of EAE in aged mice, in addition the evolution pattern between ages was different. However, NK cells from aged mice presented a higher state of activation (Table S8), so we focused on NK populations in more depth. We studied different NK cell maturation states (from more immature to more mature), and could determine that both immature NK populations were increased in non‐diseased aged mice and that more specifically, CD27^+^ immature NK cells were maintained increased along EAE in aged mice. On the contrary, M1 NK cells were decreased in non‐diseased aged mice but then increased in the inflammatory phase of EAE in this age group. M2 NK cells, which are the most mature NK cells, were found expanded in the chronic phase of EAE compared to non‐disease condition and to the inflammatory phase in both ages, but aged mice showed a decreased frequency compared to young mice, suggesting that NK maturation is impaired in aged mice (Figure 6A). The frequencies of all NK cell populations showed different evolution patterns throughout EAE progression depending on age (Table S9).

Considering that the main age‐related changes occurred in T and NK cells, we next studied the cytotoxic capacity of CD8^+^ T cells, NKT, and NK cells. CD62L is a molecule that increases upon maturation and it is associated with higher cytotoxicity and degranulation in NK cells (Peng et al. 2013). Consistent with the maturation status of NK cells, frequencies of cytotoxic NK cells were reduced in non‐diseased aged mice and in the inflammatory phase of EAE in this age group (Figure 6B), whereas non‐diseased aged mice presented decreased frequency of cytotoxic NKT cells compared to young non‐diseased mice (Figure 6C). The evolution pattern of both cytotoxic NK and NKT cells was different between ages along EAE (Table S10). In fact, the frequency of aged NK cells producing perforin and granzyme B was found decreased in both phases of EAE, whereas CD8^+^ T cells producing perforin and granzyme B were reduced only in the inflammatory phase of EAE (Figure 6D). The changes over time (evolution pattern) observed in cytotoxic CD8 T cells (Tc), NK, and NKT cells producing perforin and granzyme B were different between ages along EAE (Table S10). On the contrary, the degranulation capacity, by means of CD107a expression, of CD8^+^ T cells, NKT, and NK cells did not change in non‐diseased mice between ages but was found to increase during the inflammatory phase of EAE in aged mice (Figure 6E,F). Additionally, in the chronic phase of EAE, CD107a expression increased in aged NK cells (Figure 6G) and showed a different degranulation pattern along the disease (Table S11).

Altogether, our results from the peripheral immune response suggest that the immunopathogenesis and subsequent exacerbation of EAE observed in aged mice might be driven by a shift in naïve/memory T cells, a pro‐inflammatory Th1 skewed response, and an increase in highly suppressive Treg cells, accumulation of T cells with exhausted phenotype and the capacity of B cells to present antigens. Together with adaptive immunity, the aged peripheral immune response in EAE might be influenced by impaired NK cell maturation and cytotoxicity but enhanced degranulation.

### 2.4 Aged Splenocytes Exhibit Enhanced Antigen‐Specific Cytokine Production in EAE

**FIGURE 7:**
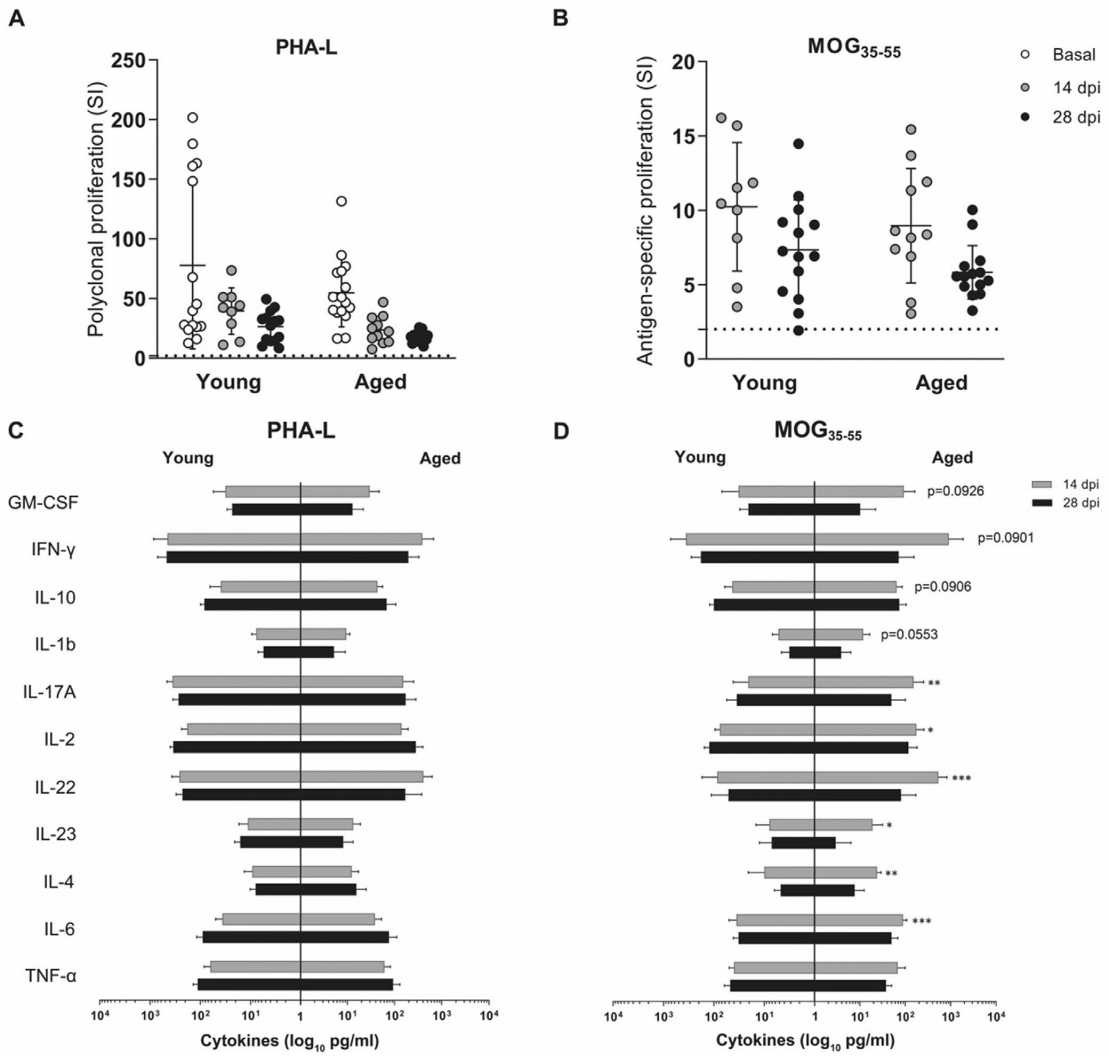
Splenocyte proliferative capacity is not altered but exhibit higher cytokine production in EAE with age. (A, B) Polyclonal (A) and MOG‐specific (B) splenocyte stimulation at basal (non‐immunized mice), 14 and 28 dpi in young and aged mice. The dotted line indicates a SI value of 2. Data represent two independent experiments with *n* = 15 (basal), *n* = 9 (14 dpi), and *n* = 14 (28 dpi) for young mice and *n* = 16 (basal), *n* = 11 (14 dpi), and *n* = 14 (28 dpi) for aged mice. (C, D) Cytokine production by polyclonal‐stimulated (C) and MOG‐stimulated (D) splenocytes at 14 dpi and 28 dpi in young and aged mice. Data represent an individual experiment with *n* = 6 (14 dpi) and *n* = 6 (28 dpi) for young mice and *n* = 6 (14 dpi) and *n* = 8 (28 dpi) for aged mice. Only incident mice that reached the endpoint were included in the analysis. Variables were analyzed using a two‐way ANOVA test for A and B and a GLIMMIX model for C and D and statistical significance correction for multiple comparisons was performed with Bonferroni adjustment. Data are expressed as the mean ± SD. *p < 0.05; **p < 0.01; ***p < 0.001. MOG, myelin oligodendrocyte glycoprotein; PHA‐L, phytohaemagglutinin‐L; SI, stimulation index.

To further define the peripheral immune response in EAE, we studied splenocyte proliferation capacity and cytokine production of antigen‐specific and polyclonal stimulated splenocytes. No differences were observed between ages in EAE, either in polyclonal (Figure 7A) or antigen‐specific (Figure 7B) proliferation capacities. Aged splenocyte supernatants obtained after polyclonal stimulation showed no differences in cytokine production (Figure 7C), however, MOG‐specific stimulation resulted in an increase in IL‐17a, IL‐2, IL‐22, IL‐23, IL‐4, and IL‐6 and a tendency to increase in GM‐CSF, IFN‐γ, IL‐10, and IL‐1b production during the inflammatory phase of EAE (Figure 7D). Our results indicate that there is an increase in antigen‐specific cytokine production, especially in cytokines related to the pro‐inflammatory response, which is consistent with our previous results in the peripheral immune response analysis.

### 2.5 Peripheral Transcriptomic Profile Changes in Aged EAE

**FIGURE 8:**
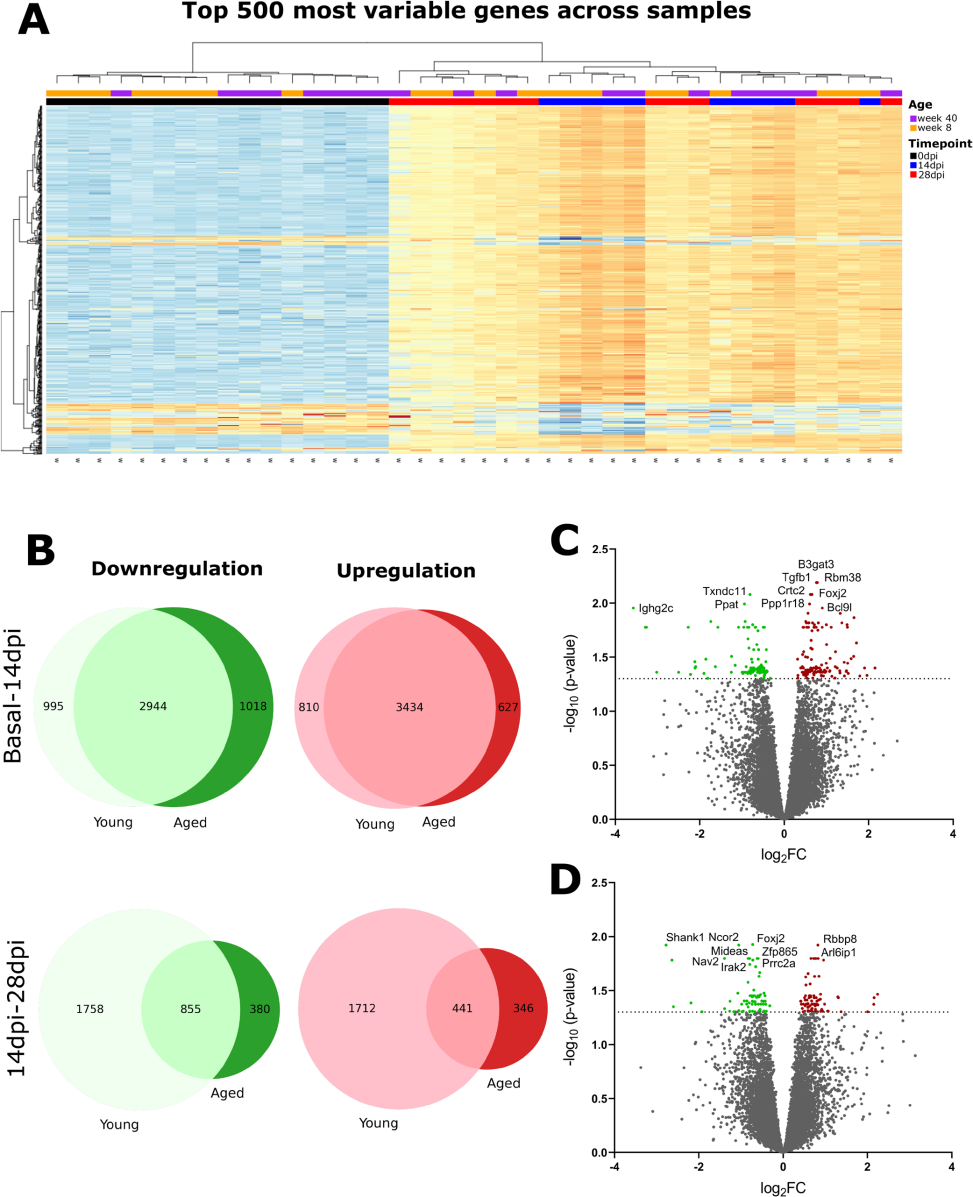
Differentially expressed genes (DEGs) highlight age‐dependent transcriptional changes during EAE progression. (A) Heatmap illustrating the top 500 most variable genes across samples, clustered by principal component analysis (PCA) of basal (non‐immunized mice), 14 dpi (days post‐induction), and 28 dpi in young (8 weeks) and aged (40 weeks) mice. (B) Venn diagrams showing the number of overlapping and unique upregulated and downregulated DEGs between young and aged mice during the transition from basal (non‐immunized mice) to 14 dpi (top row) and from 14 to 28 dpi (bottom row). (C, D) Volcano plots of DEGs comparing aged mice to young mice during the basal (non‐immunized mice) to 14 dpi (C) and 14–28 dpi (D) transitions. Highlighted genes represent those with significant differential expression (*p* < 0.05 and |log_2_FC| > 1). From basal (non‐immunized mice) to 14 dpi: 242 DEGs (130 downregulated, 112 upregulated). From 14 to 28 dpi: 144 DEGs (76 downregulated, 68 upregulated). This analysis emphasizes the reduced number of DEGs in aged mice during the chronic phase (14–28 dpi), suggesting a potential age‐dependent decline in transcriptional plasticity. Data are based on one experiment with the following sample sizes: basal (*n* = 8 young, *n* = 8 aged), 14 dpi (*n* = 5 young, *n* = 5 aged), and 28 dpi (*n* = 8 young, *n* = 6 aged). Only mice that reached the experimental endpoints were included in the analysis. DEG, differentially expressed gene; FC, fold change; PCA, principal component analysis.

To further characterize the peripheral immune response in EAE with age and find possible therapeutic targets, we obtained the transcriptomic profile of the disease time course in young and aged mice. Principal component analysis (PCA) showed sample clustering by no disease (basal) and EAE time points rather than by age (Figure S3), which matches the hierarchical clustering of the most variable genes where the same pattern was observed (Figure 8A). Firstly, we compared differentially expressed genes (DEGs) defined as genes with a fold change (FC) > 1 and an adjusted *p* < 0.05 between time points, so we obtained the DEGs from the basal to the inflammatory phase and from the inflammatory to the chronic phase for each age. We found that the proportions of DEGs are similar between ages from basal to the inflammatory phase; however, they decrease almost by half with age from the inflammatory to the chronic phase of EAE in both downregulated and upregulated DEGs (Figure 8B), which indicates that the transcriptomic profile in the inflammatory and chronic phases of EAE does not change as much in aged EAE mice as it changes in young EAE mice. Next, we compared these DEGs between ages, so that we obtained the DEGs in aged mice compared to young mice from the basal to the inflammatory phase and from the inflammatory to the chronic phase (Figure 8C,D) and obtained a gene set of 242 DEGs from basal to the inflammatory phase (Table S12) and 144 DEGs from the inflammatory to the chronic phase (Table S13), from which we performed our gene set enrichment analysis (GSEA).

**FIGURE 9:**
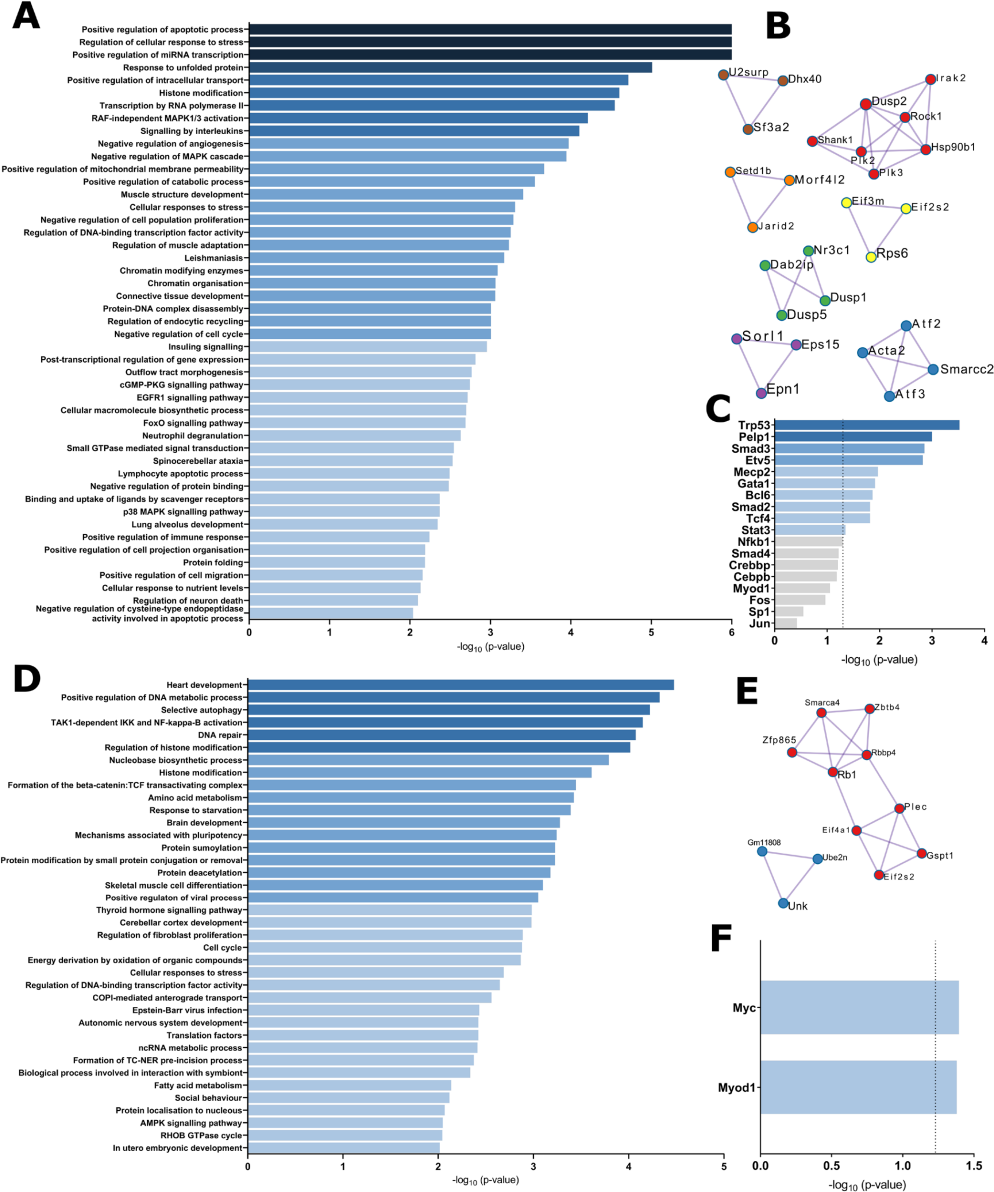
GSEA identified age‐related pathway, protein interaction, and transcriptional regulation enrichment along EAE. (A) Pathway, (B) protein interaction, and (C) transcriptional regulation enrichment analyses of aged mice compared to young mice from basal (non‐immunized mice) to 14 dpi. (D) Pathway, (E) protein interaction, and (F) transcriptional regulation enrichment analyses of aged mice compared to young mice from 14 to 28 dpi. Data represent an individual experiment with *n* = 8 (basal), *n* = 5 (14 dpi), and *n* = 8 (28 dpi) for young mice and *n* = 8 (basal), *n* = 5 (14 dpi), and *n* = 6 (28 dpi) for aged mice. Only incident mice that reached the endpoint were included in the analysis. Protein interaction enrichment analysis is based on physical interactions with at least one other protein. The enrichment analyses have been carried out using the following ontology sources: GO Biological Processes, KEGG Pathway, Reactome Gene Sets, CORUM, PaGenBase and WikiPathways STRING, BioGrid, OmniPath, InWeb_IM and TRRUST.

GSEA identified age‐related differences in pathway, protein interaction, and transcriptional regulation enrichment analyses in the EAE time course. From basal to the inflammatory phase of EAE, we found different pathways that are enriched in aged mice upon EAE induction, including *signaling by interleukins, negative regulation of cell population proliferation, negative regulation of cell cycle, positive regulation of the apoptotic process, lymphocyte apoptotic process, positive regulation of immune response*, and *positive regulation of cell migration* among the most enriched pathways in aged mice, which are in line with our previous results as we found age‐related changes in inflammatory infiltrates, cells of the adaptive immunity and cytokine production. Other enriched pathways found that could have a role in the peripheral immune response with age are *response to unfolded protein, protein folding, insulin signaling, RAF‐independent MAPK1/3 activation, negative regulation of MAPK cascade, p38 MAPK cascade*, *histone modification, chromatin modifying enzymes, chromatin organization*, and *positive regulation of miRNA transcription* (Figure 9A; Table S14). A total of seven enriched protein networks were identified in aged mice (Figure 9B), which are involved in the regulation of MAPK cascade and miRNA transcription and metabolism. The most likely transcription factors involved in the regulation of DEGs with age were *Trp53, Pelp1, Smad3, Etv5, Mecp2, Gata1, Bcl6, Smad2, Tcf4*, and *Stat3* (Figure 9C; Table S16).

From the inflammatory to the chronic phase of EAE, we found the *cell cycle* among the most enriched pathways in aged mice. Other enriched pathways found that could have a role in the peripheral immune response with age are *regulation of histone modification, histone modification, ncRNA metabolic process, selective autophagy, TAK1‐dependent IKK and NF‐kappa‐B activation, fatty acid metabolism, positive regulation of viral process*, and *Epstein–Barr virus (EBV) infection* (Figure 9D; Table S15). Compared to the first phase of the disease, only two protein networks were identified in aged mice (Figure 9E), which were involved in DNA repair and TAK1‐dependent IKK and NF‐kappa‐B activation. The most likely transcription factors involved in the regulation of DEGs with age were *Myod1* and *Myc* (Figure 9F; Table S17), meaning that they could have a key role in the regulation of the genes involved in the latest phase of the immunopathogenesis of EAE when major age‐related changes took place.

## 3 Discussion

Herein, we focused our interest on the characterization of the differential immunopathogenic mechanisms that take place with age in EAE, a well‐established experimental model of MS, to provide evidence of the impact of immunosenescence on the immunopathogenesis of the disease. EAE is a widely used animal model to study MS. The utility of the EAE model lies in its ability to recapitulate key pathological and immunological features of MS. These include blood–brain barrier disruption, infiltration of immune cells into the CNS, demyelination, axonal damage, and progressive neurodegeneration (Constantinescu et al. 2011; Lassmann and Bradl 2017). EAE also provides a versatile platform for studying various MS subtypes and their pathogenesis, as it can be induced in different mouse strains using specific myelin antigens, resulting in phenotypes that mimic the relapsing–remitting, primary progressive, or secondary progressive forms of MS (Robinson et al. 2014). These features allow researchers to explore distinct disease mechanisms and test therapeutic approaches in a controlled and reproducible manner. While EAE is not an exact replica of MS due to inherent species differences and the absence of spontaneous disease onset, it serves as a valuable bridge to understanding MS. Many findings derived from the EAE model, such as the role of Th1 and Th17 cells, Treg cells, and the involvement of B cells in CNS autoimmunity, have been confirmed in human MS studies (Fletcher et al. 2010; Hemmer, Kerschensteiner, and Korn 2015). Furthermore, the model enables preclinical testing of therapies, some of which have translated successfully to clinical use in MS patients, including natalizumab and fingolimod (Steinman and Zamvil 2006).

Age shapes the clinical outcome of MS. Patients with PPMS normally have a later disease onset than those with RRMS, the majority of whom develop SPMS over time (Scalfari et al. 2011). In the present study, we used 8‐week‐old mice (20 years old in humans), when MS can appear in young adults and 40‐week‐old mice (50 years old in humans), the age around PPMS onset, and around the conversion from RRMS to SPMS. According to what is observed in MS patients, the disease was more severe in aged mice, presenting a higher degree of disability. Similarly, other studies also reported that EAE was more severe with age (Atkinson et al. 2022; Lee et al. 2021; Wang et al. 2022); however, the molecular and cellular mechanisms underlying such age‐related differences were not studied and are still unclear.

Likewise other studies (Atkinson et al. 2022), we demonstrated that the aging process in EAE enhances neuroinflammation and neurodegeneration of the CNS. Our results indicate that aged EAE mice compared to their younger counterparts present an increase in macrophage and T cell recruitment to the damaged area, as well as more reactive microglia and astroglia, demyelination, and axonal damage. Indeed, these histopathological findings are in agreement with the more severe EAE clinical outcome observed in aged EAE mice compared to young EAE mice. On the other hand, although aged EAE mice presented higher microglia/macrophage reactivity in the chronic phase of the disease, protective *Mrc1*, and *Chi3L3* genes were also upregulated with age. The extensive inflammation and tissue damage present in the chronic phase of EAE in aged mice probably pushes microglia to upregulate the expression of genes involved in the resolution of the inflammatory response (*Mrc1*) (Zoller et al. 2018) and oligodendrogenesis (*Chi3L3*) (Starossom et al. 2019). However, target oligodendrocyte‐lineage cells from aged mice do not express higher levels of genes involved in remyelination, because they are not responsive to either Mrc1 and Chi3L3 or because those differences in *Mrc1* and *Chi3L3* expression are not translated into higher levels of protein.

Regarding the peripheral immune response, the main age‐related changes in EAE occurred in adaptive immunity. Aging affects the activation of both CD4^+^ and CD8^+^ T cell populations, although CD4^+^ Te cells suffered more changes. Th1 cells were initially considered to be responsible for EAE and MS immunopathogenesis, as IFN‐γ was abundant in CNS lesions (Merrill et al. 1992; Traugott and Lebon 1988) and the administration of this cytokine to MS patients exacerbated the disease (Panitch et al. 1987). However, further studies demonstrated that mice deficient in IFN‐γ (Ferber et al. 1996) or IL‐12 (Becher, Durell, and Noelle 2002) were susceptible to EAE, while mice deficient in Th17 signature cytokine IL‐23 were completely resistant to EAE (Cua et al. 2003). These new evidence moved Th17 into the focus of autoimmunity research. Indeed, Th17 cells are found in CNS lesions in EAE and IL‐17 is strongly upregulated in MS lesions (Lock et al. 2002). Looking back to our results, aging in our autoimmunity context provokes an increase of both pro‐inflammatory Th responses during the inflammatory phase. However, the Th1/Th17 ratio was increased in the inflammatory phase of EAE in aged mice. Similarly, the percentage of CD4^+^ T cells secreting the regulatory cytokine IL‐10 was found to increase in aged EAE mice, but only the ratio between Th17/Treg was decreased, meaning that there is a predominance in Th1 and Treg responses over the Th17 response in EAE with aging. Th17 cells demonstrate substantial developmental plasticity after their commitment towards Th1 cells (Lee et al. 2009) and IFN‐γ and IL‐4 negatively regulate Th17 development (Park et al. 2005), these facts may explain the higher Th1/Th17 ratio found along the disease with age. However, other possibilities based on current research on the aging process in the immune system rely on the finding that Th17/Treg ratio is decreased in centenarians, who do not develop autoimmunity or other age‐related chronic diseases that are so prevalent in the elderly. The reduced Th17/Treg ratio, together with other immune factors, is associated with a unique form of inflammaging in these very special age groups. This form of inflammaging seems to adapt to their physiological status, and thus allow them to better respond to immune stressors, rather than being associated with the development of age‐related chronic diseases. This observation supports one of the hypotheses proposed to explain why individuals over 100 years old do not develop autoimmune diseases compared to other elderly populations [as reviewed in (Anaya et al. 2024)]. In our context, we are studying healthy aged mice that are experimentally induced to develop an autoimmune condition. This suggests that the reduced Th17/Treg ratio in our old mice may be acting as a compensatory mechanism or a marker of resistance to pathogenic conditions rather than being a detrimental hallmark of EAE progression. In this scenario, evidence from human MS samples should be examined to firmly identify the decrease in the Th17/Treg ratio as a key target for controlling autoimmunity particularly in older patients.

There is an accumulation of peripheral Treg cells with aging, but the role of these cells in the aged immune system is unresolved. However, recent evidence points to the accumulation of aged Treg cells as detrimental in the context of EAE, since the proportion of polyclonal Treg cells is higher than that of antigen‐specific Treg cells (Wang et al. 2022). Our results did not show differences in peripheral Treg cells, probably because this difference is noticeable in mice older than 40‐week‐old. However, in our study, non‐immunized aged mice present some features of immunosenescence that may contribute to developing a more severe EAE clinical course: accumulation of highly suppressive Treg cells within the Treg cell population, which can be interpreted as an initial change in Treg frequency, and higher frequency of exhausted CD4^+^ T cells expressing PD‐1 and LAG‐3 (Channappanavar et al. 2009; Decman et al. 2012; Lee et al. 2016; Song et al. 2018).

Considering that B cell activation was increased with age, we measured anti‐MOG_35‐55_ IgG autoantibody production but found no differences between ages. However, our EAE model is not the most appropriate to study the humoral response, as it has been demonstrated that the autoantibodies produced in the rat MOG_35‐55_‐induced EAE model were non‐pathogenic (Marta et al. 2005). In addition to their role as antibody secretors, B cells are also antigen‐presenting cells (APCs). Recently, a single‐cell transcriptomic study identified *antigen processing and presentation* as one of the most upregulated pathways in different immune populations of aged mice, including B cells (Teo et al. 2023). Indeed, we observed an increase in MHC‐II expression on B cells during the inflammatory phase of EAE with age. Thus, our findings contribute to reinforce the statement that B cells might be more prone to antigen presentation rather than autoantibody secretion and, subsequently, activation of autoreactive CD4^+^ T cells that promote the pro‐inflammatory response in EAE with aging, leading to an increase in disease severity.

Consistent with the literature, peripheral innate immunity suffered less age‐related changes than adaptive immunity. The cells that suffered most of these age‐related changes were NK and NKT cells, especially their frequencies and functionality. Aging appears to promote changes in the distribution of NK cell subsets, with a reduction in immature and an increase in mature NK cells (Huntington et al. 2007). In contrast, our study points to an impairment in NK cell maturation with age in EAE, as the frequencies of CD27^+^ immature NK cells increased while mature M2 NK cells decreased. This impairment in the frequency of mature NK cells has already been described in peripheral tissues in healthy aged mice (Beli et al. 2014). In line with these results, we also observed a decrease in CD62L expression by NK and NKT cells, which is also critical in NK maturation. This marker is associated with a more cytotoxic profile (Peng et al. 2013), suggesting that NK cells in aged EAE mice might be less cytotoxic. This statement was later confirmed since the cytotoxic activity measured by the capacity to produce perforin and granzyme B of CD8^+^ T and NK cells was decreased in aged non‐immunized and EAE mice. Cytotoxicity of NK cells is reduced with age (Hazeldine, Hampson, and Lord 2012) rather than by MS condition (Lunemann et al. 2011). However, and contrary to what we currently describe in the MOG‐induced EAE model, perforin‐secreting NK cells are expanded in SP and PPMS patients compared to sex‐ and age‐matched healthy controls (Plantone et al. 2013). Contrarily, the degranulation capacity was increased in aged EAE mice. Since the cytotoxic capacity was reduced in aged mice, we hypothesize that the vesicle content might be empty or, alternatively, CD8^+^ T and NK cells might be degranulating other cytotoxic molecules rather than perforin and granzyme B with aging. The decrease in the cytotoxic activity of these cells could be also the result of the highly suppressive Treg cell accumulation with age, as Treg cells are capable of decreasing NK and CD8^+^ T cell cytotoxic function (Trzonkowski et al. 2006).

The transcriptomic profile of the peripheral immune response in EAE confirmed that the immunopathogenesis of the disease is different from aging. During the progression of EAE, we have differentiated two different phases: from basal to the inflammatory period that corresponds to the inflammatory phase and from the inflammatory to the chronic period that corresponds to the chronic phase. In the inflammatory phase, the most enriched pathway identified with aging was the *positive regulation of the apoptotic process* that together with *lymphocyte apoptotic process, negative regulation of cell population proliferation*, and *negative regulation of cell cycle* pathways probably could be involved in the incapacity to replenish the pool of Tn cells and the accumulation of Te cells in the periphery of aged EAE mice. The pathways *response to unfolded protein* and *protein folding* have also been found enriched in this first phase of EAE in aged mice. An increase in the biosynthesis of inflammatory mediators, as we observed in aged EAE mice, triggers endoplasmatic reticulum (ER) stress, which is caused by the accumulation of unfolded or misfolded proteins. The unfolded protein response (UPR) is activated to restore ER homeostasis and, ultimately, trigger apoptosis if the cell is suffering excessive ER stress. UPR markers have been reported to be elevated in different cell types in MS and EAE, including T cells (Mhaille et al. 2008). In addition, excessive ER stress has been associated with the aging process and contributes to an inflammatory environment (Salminen, Kaarniranta, and Kauppinen 2020). Moreover, it is well established that centenarians have a specific immune phenotype that protects them from developing autoimmunity, and good maintenance of proteodynamics has been pointed as a clue factor to be able to better respond to stressors and so delay immunosenescence‐related processes such as oxidation, cellular aging, and immune dysfunction (Anaya et al. 2024). Another enriched pathway identified has been *insulin signaling* which is an essential pathway for the correct functioning of the adaptive immune system, including the activation and cytokine production of CD4^+^ T cells and the cytotoxic capacity of CD8^+^ T cells. Indeed, blocking the insulin receptor results in an amelioration of EAE clinical outcome (DiToro et al. 2020). *RAF‐independent MAPK1/3 activation*, *negative regulation of MAPK cascade*, and *p38 MAPK cascade* were identified as key pathways with aging in EAE. MAPKs contribute to disease aggravation, specifically, activation of p38 MAPK in CD4^+^ T cells is essential for IL‐17 production and its blockade suppresses EAE development (Noubade et al. 2011). There is a great number of emerging evidence that epigenetic modifications may alter cellular functions and confer risk for MS. Several studies on EAE point to the potential use of histone deacetylase (HDAC) inhibitors (Camelo et al. 2005; Ge et al. 2013; Zhang et al. 2012) and activators (Zhang et al. 2009) as transcriptional modulators to ameliorate MS, suggesting that *histone modification*, *chromatin modifying enzymes*, and *chromatin organization* pathways might also be involved in the differential clinical outcome presented with aging in EAE. Moreover, *positive regulation of miRNA transcription* pathway was enriched in aged EAE mice and has been widely described as the involvement of miRNAs in autoimmunity, being miR‐155 one of the most studied in the context of EAE and MS (Murugaiyan et al. 2011; Paraboschi et al. 2011). Other important pathways found dysregulated with aging in EAE were *signaling by interleukins, positive regulation of immune response*, and *positive regulation of cell migration*, which is in line with the observed increase in cytokine production profile and subsequent CNS inflammatory environment, probably as a result of the inflammaging process.

In the chronic phase of the disease, there are pathways such as *regulation of histone modification, histone modification, ncRNA metabolic process*, and *cell cycle* that stay enriched in time, suggesting they might have a major role in the severity of the disease in aged mice. *Selective autophagy* is a key enriched pathway as it has already been linked to MS. Specifically, the expression of the autophagy‐related gene 5 (Atg5) is increased in peripheral T cells during an active relapse in RRMS patients and higher expression of the gene correlates with disease severity in EAE mice (Alirezaei et al. 2009), deciphering a connection between autophagy and MS. On the other hand, Atg5 is involved in the generation of modified self‐peptides in APCs. Autophagy constitutes an efficient antigen‐processing pathway by which proteins are delivered to MHC‐II molecules for their presentation, indicating a possible involvement of autophagy in the activation of the autoimmune response in EAE with aging (Ireland and Unanue 2011). The NFkB transcription factor plays a central role in the regulation of inflammation and has already been described to participate in the development of EAE and MS (Jia et al. 2011), suggesting that the *TAK1‐dependent IKK and NF‐kappa‐B activation* pathway might be in part responsible for the worsening of EAE with aging. *Fatty acid metabolism* pathway has also arisen as altered in EAE with aging. Nonetheless, fatty acid metabolism is crucial during T cell differentiation and there is a great amount of evidence that demonstrates that Th17 and Treg require different metabolic pathways for their correct differentiation. While Th17 development depends on the *de novo* synthesis of fatty acids, Treg development relies on the uptake and oxidation of external fatty acids (Berod et al. 2014). Changes on this metabolic pathway could be involved in the increase in Th17 and Treg cytokine production observed in EAE with age. *Positive regulation of viral process* and *EBV infection* pathways are two of the most enriched in aged EAE mice. This is relevant since EBV has been recently proposed as a trigger for MS (Bjornevik et al. 2022) and alterations in the response to EBV and other viruses in aged individuals might have an impact on the development of the disease. In addition, hyperstimulation of the immune system by, for example, viral infections or exposure to chemicals is intimately linked to the breakdown of immune tolerance, and thus to autoimmunity. However, it has not yet been established whether a low exposition to environmental factors or protection to hyperstimulation of the immune system due to genetic and/or epigenetic background of the individuals, or both, are more relevant to control tolerance breakdown in elderly (Anaya et al. 2024).

We identified 10 enriched transcription factors in the first phase of the disease with age, of which *Trp53, Smad3, Mecp2, Bcl6, Tcf4*, and *Stat3* have already been described to be involved in the peripheral immune response of MS patients (Ahmad et al. 2022; Asashima et al. 2022; Deng et al. 2005; Horjus et al. 2022; Li et al. 2020; Li et al. 2019). It is noteworthy that only *Myc* and *Myod1* were identified in the second phase of the disease. Although *Myc* has been previously studied in EAE and MS (International Multiple Sclerosis Genetics et al. 2011; Kunkl et al. 2019; Webb et al. 2019), to date, there are no studies describing the role of this critical transcription factor in the context of aging autoimmunity. Therefore, our analysis revealed *Myc* as a possible key transcription factor in the chronic phase of EAE, when major age‐related changes took place.

Nonetheless, the present study presents certain limitations. The principal limitation is the challenge to extrapolate the findings described in the immune system of aged EAE mice to patients with MS with an age around 50 years old; while the MOG‐induced EAE model provides valuable insights into immune‐mediated mechanisms of MS, it does not fully replicate the complexity of the human disease, including its heterogeneity in clinical progression. In addition, mice are in a controlled experimental environment that misrepresents the multifactorial influences on MS development and progression in humans, including the absence of environmental triggers. Prolonged hyperstimulation of the immune system due to repeated infections as well as exposure to chemical substances may have a prominent role in the development of autoimmunity in the elderly (Anaya et al. 2024), although there are authors that postulate that immune resilience is independent of inflammatory stress, instead it has to do with the capacity to recover the immune function to optimal or pre‐exposure levels (Ahuja et al. 2023); although we are missing these triggers when using the animal model, since mice are in a controlled environment and thus have a low‐grade (or nil) exposome, we cannot be sure the impact they would have in the progression of EAE in aged mice. Nevertheless, the use of the EAE model in this context provides a well‐established platform for dissecting age‐related immunological mechanisms, offering critical insights into how aging influences immunopathogenesis at the peripheral and local level at different phases of the disease, which are difficult to achieve with human studies alone. It is also important to highlight that we observed differences in the immune system between young and aged mice under basal or non‐disease conditions. However, these findings do not negate the significance of the age‐related changes identified during EAE. Instead, these changes emphasize how immunological mechanisms activated during pathological processes such as EAE may amplify or modulate preexisting age‐associated alterations, which are critical to understanding the impact of aging on MS immunopathogenesis.

These limitations notwithstanding, our study provides a foundational framework for understanding age‐related immunological mechanisms in MS and their contribution to disease severity and progression. Future studies should aim to validate these findings in human cohorts to bridge the gap between preclinical and clinical research. Such efforts will be crucial to determine the translational potential of the identified mechanisms and their impact on MS progression and treatment efficacy in older patients. Additionally, this study opens new avenues for exploring the intricate interplay between aging, immune modulation, and neurodegeneration, emphasizing the necessity of integrating age as a critical variable in MS research.

## 4 Conclusions

The present study comprehensively describes the age‐related changes in the clinical outcome, peripheral and local immune response, and neurodegeneration environment that take place in different phases of EAE in an aging context. Differences in EAE outcome with age may be a consequence of a mixture of factors occurring in the periphery and locally in the CNS. We identified major age‐related changes in the adaptive rather than in the innate immune response in the periphery: a shift in naïve/memory T cell ratio, a pro‐inflammatory Th1 skewed response, an increase in highly suppressive Treg cells, accumulation of exhausted CD4^+^ T cells, increased capacity of B cells to present antigens, and a decrease in NK cell maturation and cytotoxicity. We also report changes in the inflamed and damaged CNS in aged EAE mice, which present an increased inflammatory and neurodegenerative environment, clearly contributing to worsening disease outcomes. Finally, the transcriptomic profile study of the peripheral immune system opens the field to explore potential targets in the EAE model for the development of new therapies specifically indicated for MS patients over 50 years old, who often present increased disability.

## 5 Material and Methods

### 5.1 Experimental Model

Females were used in this study due to their higher susceptibility to the disease, the significant impact of hormonal differences on immune responses, and the greater clinical relevance of findings to human MS, which predominantly affects women (Federation 2020).

C57BL/6JRccHsd 8‐week‐old (20 years old in humans) and 40‐week‐old (50 years old in humans) (Dutta and Sengupta 2016) female mice (Envigo, Horst, Netherlands) were housed under standard light and climate‐controlled conditions and standard chow and water were provided *ad libitum*. All data presented are in accordance with the guidelines suggested for EAE publications (Baker and Amor 2012) and the *Animal Research: Reporting of* In Vivo *Experiments* (ARRIVE) guidelines for animal research (Percie du Sert et al. 2020).

### 5.2 EAE Induction and Clinical Follow‐Up

Anesthetized mice were immunized by subcutaneous injections of 100 μL PBS 1× containing 100 μg of rat peptide 35–55 of MOG_35‐55_ (Proteomics Section, Universitat Pompeu Fabra, Barcelona, Spain) emulsified in 100 μL of complete Freund's adjuvant [incomplete Freund's adjuvant (IFA, F5506, Merck, Darmstadt, Germany) containing 4 mg/mL *Mycobacterium tuberculosis* H37RA (231141; BD, Franklin Lakes, NJ, USA)]. At 0 and 2 days post‐immunization (dpi), mice were intravenously injected with 250 ng of pertussis toxin (P7208; Merck). Mice were weighed and examined daily for neurological signs in a blinded manner using the following criteria: 0, no clinical signs; 0.5, partial paresis of tail; 1, paralysis of whole tail; 2, mild paraparesis of one or both hind limbs; 2.5, severe paraparesis or paraplegia of hind limbs; 3, mild tetraparesis (mild in hind limbs); 3.5, moderate tetraparesis (moderate in hind limbs); 4, tetraparesis (severe in hind limbs); 4.5, severe tetraparesis; 5, tetraplegia; and 6, death (Edo et al. 2022). If weight loss was > 15%, mice received subcutaneous administration of 0.5 mL of 10% glucose, and endpoint criteria were defined as a weight loss > 30% or a clinical score of 5.

Mice were euthanized before the induction of EAE (basal condition), at 14 dpi (inflammatory phase) and 28 dpi (chronic phase) using carbon dioxide > 70% and spinal cord, spleen, and serum were collected.

### 5.3 Histopathological Analysis

Coronal sections of the lower spinal cord embedded in paraffin were used to analyze inflammatory infiltrate by hematoxylin and eosin (H&E) staining and images were acquired using a NanoZoomer slide scanner and NDP.view2 visualization software (Hamamatsu Photonics, Hamamamtsu, Japan). Demyelination (MBP staining), axonal damage (non‐phosphorilated heavy chain neurofilaments, SMI32 staining), reactive microglia/macrophages ( *Lycopersicon esculentum* , LEA staining), reactive astroglia (GFAP staining), and infiltrating T cells (CD3 staining) were performed (see Table S18 for primary and secondary antibody details) as previously described (Calvo‐Barreiro et al. 2020). Acquisition of immunofluorescent images and quantifications were performed as described elsewhere (Calvo‐Barreiro et al. 2020). For each mouse and staining condition, two mosaic tiles of the thoracic spinal cord separated by 300 μm were analyzed in a blinded manner.

### 5.4 RNA Isolation, cDNA Synthesis, and qPCR Analysis

Total RNA was isolated from the upper spinal cord using QIAzol lysis reagent (79306; Qiagen, Venlo, Netherlands) and RNeasy Mini Kit (74104; Qiagen) with on‐column DNase digestion (79254; Qiagen) and further purified with Turbo DNAse‐free Kit (AM1907; Thermo Fisher Scientific) to remove any genomic trace. All the procedures were carried out according to the manufacturer's instructions. RNA quality was analyzed by capillary electrophoresis using a Bioanalyzer 2100 (Agilent Technologies, Santa Clara, CA, USA). Next, mRNA was reverse transcribed with High‐Capacity cDNA Reverse Transcription Kit with RNase Inhibitor (4368814; Thermo Fisher Scientific), according to the manufacturer's instructions. Primers for *Il1b* (Mm00434228_m), *Il6* (Mm00446190_m1), *Mbp* (Mm01266402_m1), *Gfap* (Mm01253033_m1), *Chi3l1* (Mm00801477_m1), *Aif1* (Mm00479862_g1), *Mrc1* (Mm01329362_m1), *Chi3l3* (Mm00657889_mH), *Snap25* (Mm00456921_m1), *Gap43* (Mm00500404_m1), *Pdgfra* (Mm00440701_m1), *Nos2* (Mm00440502_m1), and the housekeeping gene *Gapdh* (Mm99999915_g1), as well as TaqMan Gene Expression Master Mix (4369016; Thermo Fisher Scientific) were used to perform qPCR using a 7900HT Fast Real‐Time PCR System (Thermo Fisher Scientific), according to the manufacturer's instructions. The relative level of gene expression was calculated using the 2^−∆∆CT^ method (Livak and Schmittgen 2001). The expression of the housekeeping gene (*Gapdh*) was used for normalization and the expression of the gene of interest (*Il1b, Il6, Mbp, Gfap, Chi3l1, Aif1, Mrc1, Chi3l3, Snap25, Gap43, Pdgfra*, and *Nos2*) in the basal condition was used as a calibrator. Analyses were performed with SDS 2.4 software (Thermo Fisher Scientific) and any sample with a quantification cycle value greater than 35 was considered a non‐amplified sample (Bustin et al. 2009).

### 5.5 Splenocyte Proliferation Assay

Splenocytes were isolated and freshly seeded in a complete X‐VIVO 15 medium (Lonza, BE02‐060F) as previously described (Calvo‐Barreiro et al. 2020). Splenocyte cultures were stimulated with 5 μg/mL MOG_35‐55_ (antigen‐specific stimulation), 5 μg/mL *phytohaemagglutinin‐L* (PHA‐L, L2769, Merck) (polyclonal stimulation), or with no stimulus as a control condition. After 54 h in vitro, supernatants were harvested and stored at −80°C to further determine the cytokine secretion profile. At the same time, 1 μCi of [^3^H]‐thymidine (NET027Z001MC; PerkinElmer, Waltham, MA, USA) was added to each well. Splenocyte cultures were maintained under the same conditions for an additional 18 h (Calvo‐Barreiro et al. 2020). Splenocyte cultures were transferred into glass fiber filters using a FilterMate Universal Harvester (C961961; PerkinElmer) and embedded in Optima Gold F scintillation liquid (6013171; PerkinElmer) before measuring the incorporated radioactivity in a 1450 Microbeta scintillation counter (PerkinElmer). Results are expressed as the stimulation index (SI) of MOG_35‐55_ or PHA‐L conditions (Calvo‐Barreiro et al. 2020). Samples with a SI > 2 were considered to proliferate.

### 5.6 Cytokine Production Profile Assay

Cytokine secretion profile (GM‐CSF, IFN‐γ, TNF‐α, IL1b, IL‐2, IL‐4, IL‐6, IL‐10, IL‐12p70, IL‐17A, IL‐21, IL‐22, and IL‐23) was assessed in the supernatants of PHA‐L and MOG_35‐55_ stimulated splenocytes with a ProcartaPlex Multiplex Immunoassay (Thermo Fisher Scientific), according to the manufacturer's instructions. Samples were acquired in a Luminex Magpix instrument (Thermo Fisher Scientific) and data were analyzed with ProcartaPlex Analyst software (Thermo Fisher Scientific).

### 5.7 Peripheral Immune Response Analysis

Splenocytes were stained using the corresponding combination of fluorochrome‐conjugated antibodies (Table S19). Before the staining, cells were washed with PBS 1× and incubated with the corresponding fixable viability dye (FVD) to exclude death cells. Then, cells were incubated with rat anti‐mouse CD16/32 antibody (553142, BD) to avoid unspecific staining. A total of 13 panels were designed to analyze the immune cell populations of interest.

Freshly isolated splenocytes were used for the analysis of T cell differentiation and activation status, T cell exhaustion, senescent T cells, B cells and activation status, memory B cells, DCs, neutrophils and M2 macrophages, and NK cell maturation. Pro‐inflammatory and anti‐inflammatory cytokine producing CD4^+^ and CD8^+^ T cells were determined by ex vivo stimulation of splenocytes with 50 ng/mL phorbol 12‐myristate 13‐acetate (PMA, P1585; Merck) and 1 μg/mL ionomycin (I0634; Merck) in the presence of 5 μL/2 × 10^6^ cells GolgiPlug (GP, 555029, BD) and 1.33 μL/2 × 10^6^ cells GolgiStop (GS, 554724, BD) for 6 h. For analysis of cytotoxicity on CD8^+^ T, NKT, and NK cells, ex vivo stimulation of splenocytes was performed with 50 ng/mL Recombinant Mouse IL‐2 (402‐ML; Bio‐Techne, Minneapolis, MN, USA) and 200 ng/mL Recombinant Murine IL‐15 (210–15; Thermo Fisher Scientific) for 72 h and in the presence of 5 μL/2 × 10^6^ cells GP and 1.33 μL/2 × 10^6^ cells GS for the last 6 h (Fehniger et al. 2007). For analysis of degranulation on CD8^+^ T, NKT, and NK cells, ex vivo stimulation of splenocytes was performed with 50 ng/mL PMA and 1 μg/mL ionomycin in the presence of 5 μL/2 × 10^6^ cells GP and 1.33 μL/2 × 10^6^ cells GS for 6 h and CD107a was added during cell stimulation to maximize the detection of the antigen (Alter, Malenfant, and Altfeld 2004). Nuclear FoxP3 staining was performed with the FoxP3/Transcription Factor Staining Buffer Set (00‐5523‐00; Thermo Fisher Scientific) and intracellular staining of IFN‐γ, IL‐17, IL‐10, IL‐4, CD206, perforin, and granzyme B was performed using the Cytofix/Cytoperm kit (554714, BD). Samples were acquired in a CytoFLEX flow cytometer and data were analyzed with CytExpert 2.4 software (Beckman Coulter, Brea, CA, USA). T cell differentiation and activation status, NK cell maturation, and CD8^+^ T, NKT, and NK cytotoxicity were further represented using uniform manifold approximation and projection (UMAP) with FlowJo 10.8 software (BD).

### 5.8 Flow Cytometry Analysis of Peripheral Immune Populations

Based on the T cell differentiation process, we studied the frequency of different T cell populations present in the spleen (Villegas‐Mendez et al. 2016): activated T cells (CD3^+^CD4^+^/CD8^+^CD69^+^), Tn cells (CD3^+^CD4^+^/CD8^+^CD44^−^CD62L^+^), Te cells (CD3^+^CD4^+^/CD8^+^CD44^+^CD62L^−^CD127^−^KLRG1^−^), Ttde cells (CD3^+^CD4^+^/CD8^+^CD44^+^CD62L^−^CD127^−^KLRG1^+^), Tem cells (CD3^+^CD4^+^/CD8^+^CD44^+^CD62L^−^CD127^+^KLRG1^−^), and Tcm cells (CD3^+^CD4^+^/CD8^+^CD44^+^CD62L^+^CD127^+^). Treg cells were defined as CD25^+^FoxP3^+^ in the CD3^+^CD4^+^ population, and highly suppressive Treg cells were Treg expressing CD39 (CD3^+^CD4^+^CD25^+^FoxP3^+^CD39^+^). Total NK (CD3^−^NK1.1^+^) and NKT cells (NK1.1^+^CD3^+^) were also studied. NK cell maturation states (from more immature to more mature) were analyzed as described elsewhere (Chiossone et al. 2009; Huntington et al. 2007): CD27^−^ immature NK cells (CD3^−^NK1.1^+^CD11b^−^KLRG1^−^CD27^−^), CD27^+^ NK immature cells (CD3^−^NK1.1^+^CD11b^−^KLRG1^−^CD27^+^), M1 NK cells (CD3^−^NK1.1^+^CD11b^+^KLRG1^−^CD27^+^), and M2 NK cells (CD3^−^NK1.1^+^CD11b^+^KLRG1^+^CD27^−^). CD69 (activation marker) and MHC‐II frequencies were determined with respect to CD3^−^B220^+^ B cells. Additional information on markers used for the determination of peripheral immune cell subsets can be found in Table S19.

### 5.9 Quantification of Anti‐MOG Antibody Levels

Anti‐MOG_35‐55_ IgG was measured in serum samples with SensoLyte Anti‐MOG (35–55) IgG Quantitative ELISA kit (AnaSpec, AS‐54465, Fremont, CA, USA), according to the manufacturer's instructions using a BioTek Epoch Microplate Spectrophotometer (Agilent Technologies). The coefficient of variation between sample duplicates, intraplate and interplate controls < 20% was accepted.

### 5.10 Quantification of Neurofilament Light Chain Levels

NfL levels were measured in serum with the NFL immunoassay kit (103186; Quanterix), according to the manufacturer's instructions using a fully automated ultrasensitive SIMOA HD‐1 Analyzer.

### 5.11 RNA‐Seq Study

Total RNA was isolated using RNeasy Mini Kit (74104; Qiagen) with on‐column DNase digestion (79254; Qiagen), according to the manufacturer's instructions. RNA quality and concentration were analyzed by capillary electrophoresis using a Bioanalyzer 2100 (Agilent Technologies) and 1 μg per sample was used to start the libraries. PolyA tail selection was performed with Poly(A) RNA Selection Kit V1.5 (157.96; Lexogen, Vienna, Austria) with the objective of isolating mRNA and long non‐coding genes and reducing the detection of rRNA. Then, retrotranscription into DNA libraries ready for sequencing was performed with CORALL Total RNA‐Seq Library Prep Kit (095; Lexogen). Libraries were sent for sequencing 150 bp paired‐end with a coverage of 20 million reads per sample using a Novaseq 6000 (Illumina, San Diego, CA, USA).

After removing the lowly expressed genes, a quality control was performed. Following the quality control step, normalization was performed to eliminate composition biases between libraries, so a normalization factor below one indicates that the library size will be scaled down and, conversely, a normalization factor above one scales up the library size. Once the samples were normalized, differential expression analysis between ages from basal condition to 14 dpi and from 14 to 28 dpi phases was performed using limma (Ritchie et al. 2015) and edgeR (Chen, Lun, and Smyth 2016; McCarthy, Chen, and Smyth 2012; Robinson, McCarthy, and Smyth 2010) package guidelines and following *Linear Models for Microarray and RNA‐seq Data User's Guide* (Law et al. 2014; Liu et al. 2015). Focusing on the DEGs over time in aged mice compared to young mice, an FC > 1 and an adjusted *p*‐value < 0.05 were the thresholds considered for GSEA (Subramanian et al. 2005).

### 5.12 Statistics

EAE incidence and the time to reach scores 3 and 4 were analyzed using a Log‐rank test. If two or more experimental groups were compared in several independent experiments, variables were analyzed using a two‐way ANOVA test or using GLIMMIX and MIXED generalized linear models and statistical significance correction for multiple comparisons was performed using Bonferroni adjustment. Data estimation was performed using the least squares means method assuming a normal or lognormal distribution, considering age and time point as dependent variables and experiment as an independent variable. The interaction term between dependent variables was also included and named evolution. Data were expressed as the mean ± standard deviation (SD), except for the clinical score which was represented as the mean ± standard error of the mean (SEM). Statistical significance was set at an adjusted *p* value of < 0.05. Statistical analyses were performed using SAS 9.4 (SAS Institute Inc., Cary, NC, USA) and GraphPad Prism 8.0 (GraphPad, La Jolla, CA, USA).

The statistical analysis of biological significance was performed for pathway, protein physical interaction, and transcriptional regulation enrichment analyses, using the following ontology sources from the *Metascape* database (Zhou et al. 2019): GO Biological Processes, KEGG Pathway, Reactome Gene Sets, CORUM, WikiPathways, PANTHER Pathway *STRING, BioGrid, OmniPath, InWeb_IM*, and TRRUST (Han et al. 2018).

### 5.13 Study Approval

All experiments were performed in strict accordance with European Union (Directive 2010/63/EU) and Spanish regulations (Real Decreto 53/2013; Generalitat de Catalunya Decret 214/97). The Ethics Committee on Animal Experimentation of the Vall d'Hebron Research Institute approved all procedures described in this study (protocol number: 67/18 CEEA; CEA OH/10683/1).

## Author Contributions

Conceptualization: Carmen Espejo and Herena Eixarch. Methodology: María Dema, Herena Eixarch, Mireia Castillo and Carmen Espejo. Investigation: María Dema, Herena Eixarch and Carmen Espejo. Visualization: María Dema, Herena Eixarch and Carmen Espejo. Funding acquisition: Carmen Espejo. Project administration: Carmen Espejo. Supervision: Carmen Espejo and Herena Eixarch. Writing – original draft: María Dema and Herena Eixarch. Writing – review and editing: María Dema, Herena Eixarch, Arnau Hervera, Mireia Castillo, Luisa M. Villar, Xavier Montalban and Carmen Espejo. María Dema and Herena Eixarch share the first authorship, and the first place has been designed for María Dema as the junior PhD student.

## Ethics Statement

All experiments were performed in strict accordance with European Union (Directive 2010/63/EU) and Spanish regulations (Real Decreto 53/2013; Generalitat de Catalunya Decret 214/97). The Ethics Committee on Animal Experimentation of the Vall d'Hebron Research Institute approved all procedures described in this study (protocol number: 67/18 CEEA; CEA OH/10683/1). Experiments were performed according to the ARRIVE Guidelines.

## Conflicts of Interest

MD, HE, AH, MC, and CE declare no competing financial interests. LMV has received speaking honoraria or participated in advisory boards from Biogen, Bristol Myers, Horizon, Merck, Novartis, Roche, and Sanofi‐Genzyme. XM has received speaking honoraria and travel expenses for participation in scientific meetings has been a steering committee member of clinical trials or participated in advisory boards of clinical trials in the past years with Abbvie, Actelion, Alexion, Biogen, Bristol‐Myers Squibb/Celgene, EMD Serono, Genzyme, Hoffmann‐La Roche, Immunic, Janssen Pharmaceuticals, Medday, Merck, Mylan, Nervgen, Novartis, Sandoz, Sanofi‐Genzyme, Teva Pharmaceutical, TG Therapeutics, Excemed, MSIF, and NMSS.

## Supporting information


**Figure S1.** Serum NfL analysis confirms aged‐related neurodegeneration in EAE.
**Figure S2.** Age is not associated with anti‐MOG35‐55 autoantibody production in EAE.
**Figure S3.** PCA sample clustering is mainly caused by EAE time course.

**Movie S1.** EAE clinical course is more severe in aged mice.

**Movie S2.** EAE clinical course is less severe in young mice.

**Table S1.** EAE clinical parameters are more severe with age.
**Table S2.** Age‐related changes in T cell differentiation and activation status.
**Table S3.** Age‐related changes in peripheral Th responses and Treg cells.
**Table S4.** Age‐related changes in T cell exhaustion.
**Table S5.** Age‐related changes in senescent T cells.
**Table S6.** Age‐related changes in B cells and activation status.
**Table S7.** Age‐related changes in DCs, neutrophils, and M2 macrophages.
**Table S8.** Age‐related changes in NK and NKT cells.
**Table S9.** Age‐related changes in NK cell maturation.
**Table S10.** Age‐related changes in cytotoxicity of CD8^+^ T, NKT, and NK cells.
**Table S11.** Age‐related changes in degranulation of CD8^+^ T, NKT, and NK cells.

**Table S12.** Age‐related changes in DEGs from the basal to the inflammatory phase of EAE.
**Table S13.** Age‐related changes in DEGs from the inflammatory to the chronic phase of EAE.
**Table S14.** Age‐related changes in pathways from the basal to the inflammatory phase of EAE.
**Table S15.** Age‐related changes in pathways from the inflammatory to the chronic phase of EAE.
**Table S16.** Age‐related changes in transcription factors from the basal to the inflammatory phase of EAE.
**Table S17.** Age‐related changes in transcription factors from the inflammatory to the chronic phase of EAE.

**Table S18.** Antibodies used for spinal cord immunofluorescence stainings.
**Table S19.** Antibodies used for flow cytometry studies.

## Data Availability

The transcriptomic data have been deposited in NCBI's Gene Expression Omnibus (GEO) and are accessible through GEO Series accession number GSE227650. Further information and requests for resources and reagents should be directed to and will be fulfilled by the lead contact, Carmen Espejo (carmen.espejo@vhir.org).
