# Correction to “Disease Aggravation With Age in an Experimental Model of Multiple Sclerosis: Role of Immunosenescence”

**DOI:** 10.1111/acel.70129

**Published:** 2025-06-03

Dema M, Eixarch H, Hervera A, Castillo M, Villar LM, Montalban X, Espejo C. *Aging Cell*. 2025 May;24(5):e14491. https://doi.org/10.1111/acel.14491. PMID: 39894902.

In the Acknowledgments section, the following acknowledgment was inadvertently omitted from the final version:

“We would also like to thank Cristina Tuñí and Flomics Biotech for their support in carrying out the RNA‐Seq study.”

The corrected acknowledgment would be:

We thank Xavier Vidal and the Statistics and Bioinformatics Unit (UEB) at Vall d'Hebron Hospital Research Institute (VHIR) for their support in carrying out the statistical analysis. We would also like to thank the Biologia‐Bellvitge Unit from Scientific and Technological Centers (CCiTUB), Universitat de Barcelona, and staff Beatriz Barroso and Esther Castaño for their support and advice on UMAP representations. Finally, we would also like to thank Cristina Tuñí and Flomics Biotech for their support in carrying out the RNA‐Seq study.

We apologize for this error.

