# Supplementary material for: Disease Aggravation With Age in an Experimental Model of Multiple Sclerosis: Role of Immunosenescence
Source: Aging Cell. 2025 Feb 2;24(5):e14491. doi: 10.1111/acel.14491 (PMC12073911; doi:10.1111/acel.14491)
Supplement: Supplementary file 1 — Figure S1. Serum NfL analysis confirms aged‐related neurodegeneration in EAE. Figure S2. Age is not associated with anti‐MOG35‐55 autoantibody production in EAE. Figure S3. PCA sample clustering is mainly caused by EAE time course. [file ACEL-24-e14491-s003.docx]

***Dema et al.***

**Supplemental information**

**Table S1- EAE clinical parameters are more severe with ag**

| **Parameter** | **Young** | **Aged** | **P-value** |
| --- | --- | --- | --- |
| Incidence | 22/24 (91.75%) | 24/24 (100.0%) | 0.0343 |
| Clinical onset | 15.3 ± 3.8 | 14.0 ± 2.6 | 0.1806 |
| Maximum clinical score | 3.8 ± 0.7 | 4.4 ± 0.6 | 0.0037 |
| Clinical course (AUC) | 39.8 ± 16.9 | 56.1 ± 16.2 | 0.0017 |
| Weight loss (AUC) | 71.4 ± 147.9 | -371.9 ± 107.2 | <0.0001 |
| Time to score 3 | 20/24 (83.33%) | 24/24 (100%) | 0.0157 |
| Time to score 4 | 15/24 (62.5%) | 22/24 (91.75%) | 0.0750 |

EAE clinical parameters at 28 dpi in young and aged mice. Data represent three independent experiments with n=22 for young mice and n=24 for aged mice at 28 dpi. Variables were analyzed using Log-rank test for incidence, time to score 3 and 4 and MIXED model for clinical onset, maximum clinical score, clinical course (AUC) and weight loss (AUC) and statistical significance correction for multiple comparisons was performed with Bonferroni adjustment. Data are expressed as the mean ± SD. AUC: area under the curve.


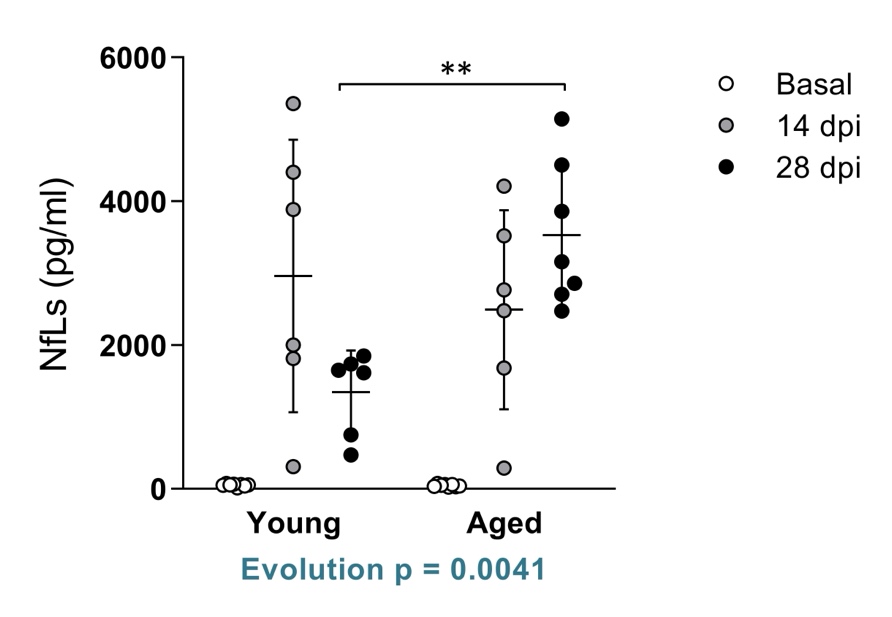


**Figure S1. Serum NfL analysis confirms aged-related neurodegeneration in EAE**

Quantification of NfL levels in serum at basal, 14 dpi and 28 dpi in young and aged mice. Data represent an individual experiment with n=8 (basal), n=6 (14 dpi) and n=6 (28 dpi) for young mice and n=8 (basal), n=6 (14 dpi) and n=7 (28 dpi) for aged mice. Only incident mice that reached the endpoint were included in the analysis. Variables were analysed using MIXED model and statistical significance correction for multiple comparisons was performed with Bonferroni adjustment. Data are expressed as the mean ± SD. **p<0.001. NfL: neurofilament light chain.


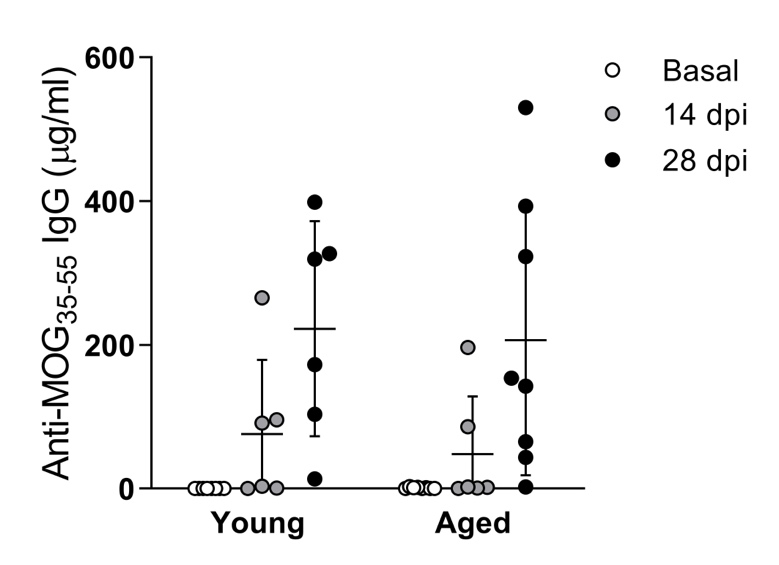


**Figure S2. Age is not associated with anti-MOG_35-55_ autoantibody production in EAE**

Quantification of anti-MOG_35-55_ IgG levels in serum at basal, 14 dpi and 28 dpi in young and aged mice. Data represent an individual experiment with n=8 (basal), n=6 (14 dpi) and n=6 (28 dpi) for young mice and n=6 (basal), n=6 (14 dpi) and n=8 (28 dpi) for aged mice. Only incident mice that reached the endpoint were included in the analysis. Variables were analysed using MIXED model and statistical significance correction for multiple comparisons was performed with Bonferroni adjustment. Data are expressed as the mean ± SD. MOG: myelin oligodendrocyte glycoprotein.


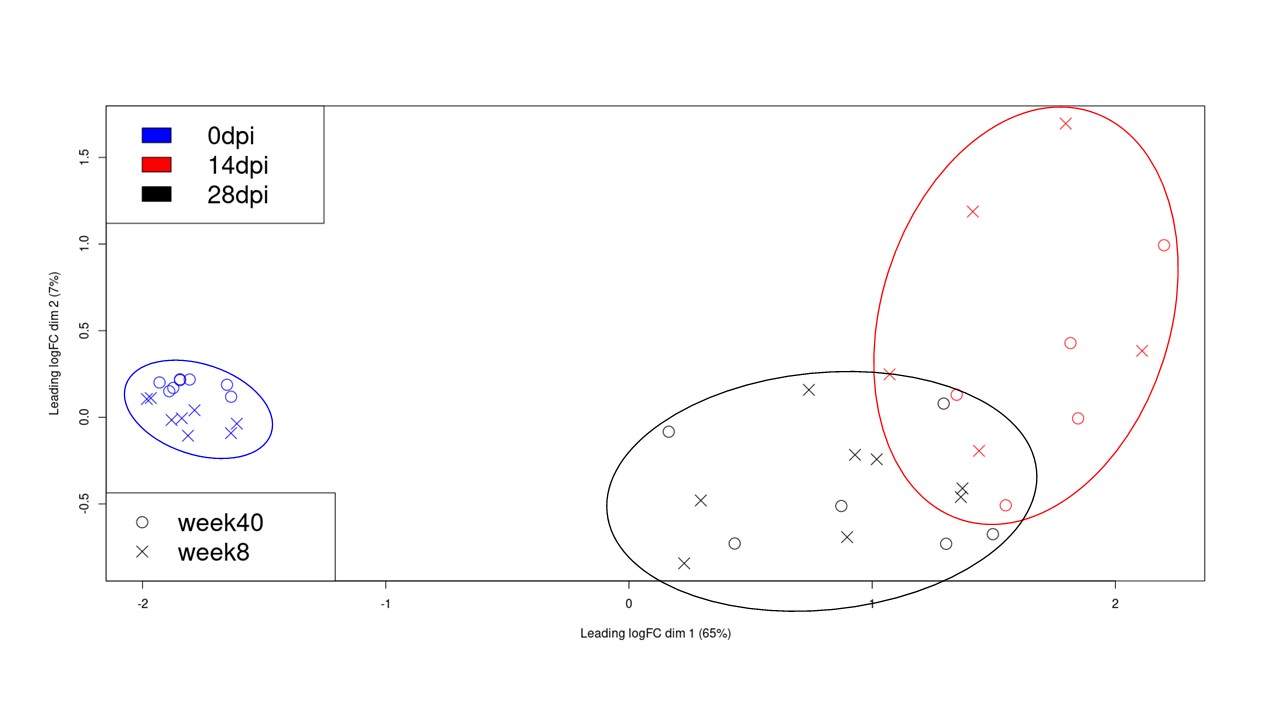


**Figure S3. PCA sample clustering is mainly caused by EAE time course**

PCA sample clustering at basal, 14 dpi and 28 dpi in young and aged mice. Data represent an individual experiment with n=8 (basal), n=5 (14 dpi) and n=6 (28 dpi) for young mice and n=8 (basal), n=5 (14 dpi) and n=8 (28 dpi) for aged mice. Only incident mice that reached the endpoint were included in the analysis. PCA: principal component analysis.
